# Ubiquitin-independent pathway regulates the RIT1-MAPK pathway in chordoma progression

**DOI:** 10.1038/s41419-025-08092-z

**Published:** 2025-10-24

**Authors:** Hui Chen, Qiujing Guan, Cheng Yang, Yang Chen, Yan Liu, Wenjie Ren, Su Chen, Lei Li, Dongxia Li, Jianguo Tang, Nanzhe Zhong

**Affiliations:** 1 Department of Trauma-Emergency & Critical Care Medicine, Shanghai Fifth People’s Hospital, Fudan University, Shanghai, China; 2 Shanghai Key Laboratory of Regulatory Biology, Institute of Biomedical Sciences, School of Life Sciences, East China Normal University, Shanghai, China; 3 Joint Center for Translational Medicine, Shanghai Fifth People’s Hospital, Fudan University and School of Life Science, East China Normal University, Shanghai, China; 4 Department of Orthopedic Oncology, The Second Affiliated Hospital of Naval Medical University, Shanghai, China; 5 Chongqing Key Laboratory of Precision Optics, Chongqing Institute of East China Normal University, Chongqing, China; 6 College of Metrology Measurement and Instrument, China Jiliang University, Hangzhou, China

**Keywords:** Bone cancer, Bone cancer

## Abstract

Chordoma is a rare, slow-growing malignant tumor originating from embryonic notochord remnants and is often found in the sacrum or skull base. It is categorized into conventional, poorly differentiated, and dedifferentiated types, with the conventional type being the most common. Owing to its location near critical structures, chordoma has a high rate of local recurrence, making new therapeutic targets essential. The proteasome system, which is responsible for degrading intracellular proteins, plays a vital role in maintaining cellular function. REGγ, a proteasome activator, mediates ubiquitin-, and ATP-independent protein degradation and is overexpressed in various cancers. However, its role in chordoma remains unexplored. Ras GTPases, including RIT1, are involved in cancer progression, and understanding their involvement in chordoma could provide therapeutic insights. This study identified REGγ as a potential therapeutic target for chordoma. REGγ was found to be upregulated in chordoma, and high REGγ expression was correlated with poor clinical outcomes. It promotes cell proliferation and migration, and inhibits apoptosis, while influencing osteoclast differentiation. Mechanistically, REGγ regulates chordoma progression through the ubiquitin- and ATP-independent degradation of RIT1, which modulates the RIT1-MAPK pathway. Inhibition of RIT1 in REGγ-knockdown cells and patient-derived organoids alleviated these effects, suggesting that targeting REGγ may be a promising strategy for chordoma treatment.

## Introduction

Chordoma, a rare primary malignant tumor of the axial skeleton, is thought to originate from the malignant transformation of embryonic notochord remnants and is a slow-growing, low-grade malignant tumor that may take years or decades to become noticeable. Its location in the spine or skull base can lead to pressure on nearby nerves and structures, causing various symptoms. Approximately 50% of cases occur in the sacrococcygeal region, 35% in the cranial base, and 15% in the mobile spine [1–4]. Chordoma is pathologically divided into three distinct categories: conventional, poorly differentiated, and dedifferentiated, with the majority of cases being the conventional type [5–8]. Surgical resection is the primary treatment for chordoma. However, because of its unique growth location (often closely related to critical bone and neural structures) and strong local invasiveness of the tumor, up to 50% of chordoma patients experience postoperative local recurrence, and 30–40% of patients with progressive disease develop metastasis [9]. Therefore, new therapeutic targets for chordoma are urgently needed.

The proteasome system degrades more than 80% of intracellular proteins [10], maintaining cellular function. Disruption of this system can cause metabolic dysfunction, leading to conditions such as inflammation and cancer [11, 12]. The proteasomal degradation system is divided into ATP-ubiquitin-dependent and ATP-ubiquitin-independent pathways on the basis of different activators [13]. The proteasome activator PSME3 (REGγ), first identified as the Ki antigen in systemic lupus erythematosus autoantibodies, belongs to the REG (11S) family and mediates ubiquitin- and ATP-independent protein degradation [14, 15]. REGγ targets proteins such as SRC-3, p21, p53, p16, CK1δ, and SirT1, influencing various diseases [16–21]. REGγ is highly expressed in many types of cancers, including colon, lung, gastric, and kidney cancers, and plays a critical role in tumor progression [16, 22–24]. However, its role in chordoma development remains unexplored and requires further investigation.

Ras GTPases are well-known for their oncogenic potential in human cancers, driving extensive research on the signaling networks and mechanisms underlying disease since 1982 [25–27]. The Ras family includes more than 150 human Ras-related GTPases, many of which are conserved across species and offer insights into cellular processes [27]. Among these Ras-related GTPases, RIT1, identified over 20 years ago [28], has been linked to Noonan syndrome and cancers [29–35]. Given the established role of Ras GTPases in tumorigenesis, further exploration into the specific mechanisms by which RIT1 contributes to chordoma could reveal valuable therapeutic targets for this challenging malignancy.

In this study, we identified REGγ as a potential therapeutic target for chordoma. Our findings revealed that REGγ is upregulated in chordoma and that its expression is inversely correlated with poor clinical outcomes. Furthermore, we demonstrated that REGγ promotes the proliferation of chordoma cells, inhibits their apoptosis, and enhances their migration. Notably, the conditioned medium from chordoma cells with REGγ inhibition inhibited the osteoclast differentiation of bone marrow-derived macrophages (BMMs), suggesting that the occurrence and development of chordoma are accompanied by abnormal activation of osteoclasts. Mechanistically, we revealed that REGγ regulates the occurrence and development of chordoma via ubiquitin- and ATP-independent protein degradation of RIT1. In *REGγ*-knockdown cells, inhibiting RIT1 expression can mitigate the reduced cell proliferation, increased apoptosis, and reduced migration caused by *REGγ* knockdown. It also alleviates the inhibitory effect of conditioned medium from *REGγ*-knockdown cells on the differentiation of BMMs into osteoclasts. Further investigation revealed that REGγ promotes the development and progression of chordoma via regulating the RIT1-MAPK pathway. These findings reveal that REGγ regulates the RIT1-MAPK pathway in chordoma progression and provide new insights for targeted therapy of chordoma.

## Methods

### Reagents

RNAiso Plus (catalog number 9109, Takara), ChamQ Universal SYBR qPCR Master Mix (catalog number Q711-03, Vazyme), Cell Counting Kit-8 (catalog number AC11L054, Shanghai Life-ilab Biotech), Annexin V-APC/PI Kit (catalog number AP107, Multi Sciences), Leukocyte Acid Phosphatase (TRAP) Kit (catalog number 387A, Sigma), anti-REGγ antibody (catalog number Q711-03, abcam; catalog number 14907-1-AP, Proteintech), anti-β-Actin antibody (catalog number M177-3, MBL), anti-PARP antibody (catalog number 9542, CST), anti-RIT1 antibody(catalog number AP51476, Abcepta), anti-Erk antibody (catalog number 9102, CST), anti-p-Erk antibody (catalog number 9101, CST), anti-JNK antibody (catalog number 9252, CST), anti-p-JNK antibody (catalog number 9251, CST), anti-p38 antibody (catalog number 9212, CST), anti-p-p38 antibody (catalog number 9211, CST), anti- ARRDC5 antibody (catalog number abs101484, Absin), anti-CSNK2A1 antibody (catalog number 10992-1-AP, Proteintech), anti- Brachyury antibody (catalog number 81694, CST), Matrigel (catalog number 356255, Corning), red blood cell lysis buffer (catalog number P0013D, Beyotime), RIPA lysis buffer (catalog number C3702, Beyotime), and organoid medium (catalog number PRS-LCM-3D, Precedo).

### Cell culture

Chordoma cell lines U-CH1 (catalog number CTCC-001-0358) and MUG-Chor1 (catalog number CTCC-001-0474) were purchased from Meisen Chinese Tissue Culture Collections (Meisen CTCC), cultured in Dulbecco’s Modified Eagle’s medium (DMEM) supplemented with 10% fetal bovine serum, and incubated at 37 °C with 5% CO_2_.

### Quantitative real-time PCR (qRT-PCR)

The assay was performed as previously described [36]. Total RNA was extracted from chordoma tissues or cells via TRIzol (TAKARA). Then, 5× HiScript II qRT SuperMix (Vazyme, R222-01) was used to reverse transcribe the RNA into cDNA. Real-time fluorescence quantitative PCR was performed using 2× ChamQ Universal SYBR qPCR Master Mix (Vazyme, Q711-02) on a Roche LightCycler 480 (Roche). The experiment was repeated three times, and Fold changes were calculated by the 2^−ΔΔCt^ method. The sequences of primers used are detailed in Supplementary Table 1.

### Western blot analysis

The analysis was performed as previously described [36]. Resuspend cells or tissue samples in lysis buffer and protein separated by 10–12% SDS-gel. The separated proteins were then transferred to nitrocellulose membranes and incubated with primary antibodies overnight at 4 °C. The membrane is then incubated with the fluorescent-labeled secondary antibody at 4 °C for 1 h and analyzed in the LI-COR Odyssey Infrared Imaging System. β-Actin served as an internal control.

### Cell proliferation and clonal formation assays

U-CH1 and MUG-Chor1 cells subjected to different treatments were seeded in 96-well plates at the same density. After 1, 3, 5, and 7 days of culture, CCK8 reagent was added at a 1:10 ratio. After incubation at 37 °C for 1 h, absorbance at 450 nm was measured. For the clonal formation assay, cells were seeded in 6-well or 12-well plates, cultured at 37 °C for 10–14 days, and fixed with 4% paraformaldehyde. After the medium was discarded, the cells were washed with PBS 2–3 times, stained with 0.2% crystal violet solution, and each experiment was repeated three times.

### Transwell migration assay

Transwell chambers (8 μm) were placed in 24-well plates. After counting the successfully transfected cells, equal cell numbers were loaded resuspended in suspensions (without FBS) to the upper chamber, and DMEM medium with 10% FBS was added to the lower chamber. The cells were incubated at 37 °C for 48 h, after which the chambers were collected. The cells that passed through the membrane were fixed with 4% paraformaldehyde, washed with PBS, and stained with 0.2% crystal violet. Each experiment was repeated three times.

### Co-immunoprecipitation (CO-IP) assay

The cells were harvested and lysed with RIPA lysis buffer at 4 °C after plasmids transfection for 36 h for 30 min, followed by centrifugation (12,000 rpm, 20 min) to remove the supernatant. Specific agarose beads were added to the supernatant and incubated at 4 °C for 4 h. After incubation, the samples were washed three times with wash buffer and analyzed by Western blot analysis.

### Statistical analysis

The data were analyzed using GraphPad 8.0 software and are presented as the mean ± standard deviation. Clinical experience and statistical analysis were used to judge whether continuous variables should be categorized. Statistical significance was determined using Student’s *t* test for two groups and one-way ANOVA for multiple groups. The recurrence-free survival (RFS) and overall survival (OS) rates were analyzed using the Kaplan-Meier method. To identify independent variables correlating prognosis, the log-rank test was applied for univariate survival analysis. Then factors with *P* value ≤ 0.10 were obtained for multivariate Cox proportional hazards analysis. Markers of significance are as follows: N.S., *P* > 0.05; *P < 0.05; **P < 0.01; ***P < 0.001.

## Results

### REGγ is upregulated in chordoma and correlates with poor prognosis

**Fig. 1:**
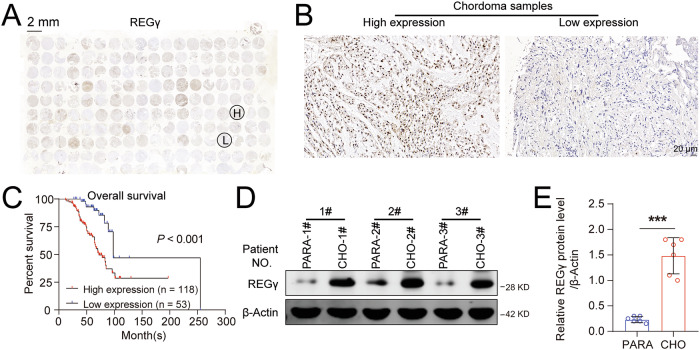
REGγ is upregulated in chordoma and correlates with poor prognosis. **A** Panoramic image of REGγ immunohistochemical (IHC) staining on the conventional chordoma tissue microarray. **B** Representative images of low and high REGγ expression in a conventional chordoma tissue microarray. Scale bar = 20 µm. **C** Kaplan–Meier survival curves of overall survival (OS) based on REGγ expression in patients with chordoma. **D** Western blot analysis was performed to evaluate REGγ and β-Actin protein levels in chordoma and matched paracancerous tissues (*n* = 6). **E** Statistical analysis results from (**D**).

REGγ is highly expressed in many types of cancer and plays a critical role in tumor progression [16, 22–24]. However, the biological function of REGγ in chordoma has not been reported. To explore whether REGγ is involved in the occurrence and development of chordoma, we first detected the expression of REGγ in conventional chordoma tissue microarray by immunohistochemical (IHC) staining. We found that REGγ is upregulated in chordoma (Fig. 1A), and that REGγ is not only negatively correlated with OS, but also negatively correlated with RFS (Figs. 1B, C, S1, and TablesS2–4), indicating that increased REGγ expression correlates with poor prognosis. Furthermore, we collected fresh samples of chordoma and matched paracancerous tissues, detected the expression of REGγ through WB analysis, and found that REGγ was significantly upregulated in chordoma (Fig. 1D, E), which was consistent with the IHC results of the chordoma tissue microarray.

### REGγ promotes proliferation and inhibits apoptosis of chordoma cells

**Fig. 2:**
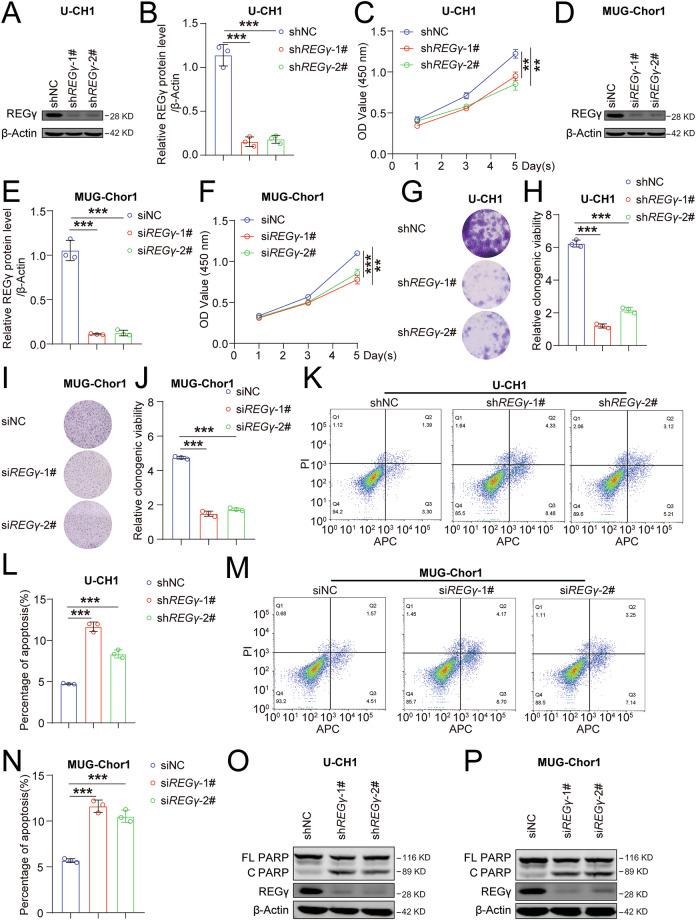
REGγ promotes proliferation and inhibits apoptosis of chordoma cells. Western blot analysis of REGγ and β-Actin expression in shNC, sh*REGγ*-1#, and sh*REGγ*-2# U-CH1 cells (**A**), with statistical analysis results shown in (**B**). **C** A CCK8 assay was used to evaluate the effect of REGγ on cell proliferation in shNC, sh*REGγ*-1#, and sh*REGγ*-2# U-CH1 cells. Western blot analysis of REGγ and β-Actin expression in siNC, si*REGγ*-1#, and si*REGγ*-2# MUG-Chor1 cells (**D**), with statistical analysis results shown in (**E**). **F** A CCK8 assay was used to evaluate the effect of REGγ on cell proliferation in siNC, si*REGγ*-1#, and si*REGγ*-2# MUG-Chor1 cells. Colony formation ability in shNC, sh*REGγ*-1#, and sh*REGγ*-2# U-CH1 cells (**G**), with statistical results in (**H**). Colony formation ability in siNC, si*REGγ*-1#, and si*REGγ*-2# MUG-Chor1 cells (**I**), with statistical results in (**J**). Cell apoptosis was assessed by flow cytometry after Annexin V-APC staining in shNC, sh*REGγ*-1#, and sh*REGγ*-2# U-CH1 cells (**K**), with statistical results in (**L**). Cell apoptosis was assessed by flow cytometry after Annexin V-APC staining in siNC, si*REGγ*-1#, and si*REGγ*-2# MUG-Chor1 cells. **M** with statistical results in (**N**). **O** Cell apoptosis was assessed by WB assay (protein levels of cleaved PARP) in shNC, sh*REGγ*-1#, and sh*REGγ*-2# U-CH1 cells. **P** Cell apoptosis was evaluated by WB assay (protein levels of cleaved PARP) in siNC, si*REGγ*-1#, and si*REGγ*-2# MUG-Chor1 cells. FL PARP: full-length PARP C PARP: cleaved PARP.

To determine whether REGγ regulates the occurrence and development of chordoma, we first established stable U-CH1 cell lines with shNC, sh*REGγ*-1#, and sh*REGγ*-2# (Figs. 2A, B and S2A). CCK8 assay showed that REGγ knockdown significantly inhibited U-CH1 cell proliferation (Fig. 2C). Similarly, silencing *REGγ* in MUG-Chor1 cells yielded the same result (Figs. 2D–F and S2B). The colony formation assay, which is commonly used to assess cell proliferation and self-renewal ability, confirmed that *REGγ* knockdown suppresses the proliferation of chordoma cells (Fig. 2G–J). These findings suggest that REGγ plays a key role in the occurrence and development of chordoma by modulating cell proliferation.

To investigate whether REGγ regulates chordoma by inhibiting apoptosis, we performed a cell apoptosis analysis. Using an Annexin V-APC/PI apoptosis detection kit combined with flow cytometry analysis, we observed that *REGγ* knockdown promoted apoptosis in U-CH1 cells (Fig. 2K, L). Western blotting revealed increased accumulation of cleaved PARP, an apoptosis marker, in *REGγ*-knockdown U-CH1 cells (Figs. 2O and S2C). We also observed the same phenomenon in MUG-Chor1 cells with *REGγ* knockdown (Figs. 2M, N and S2D). These data indicate that *REGγ* knockdown inhibits proliferation and promotes apoptosis in chordoma cells.

### REGγ promotes the metastasis of chordoma cells, and conditioned medium from REGγ-inhibited chordoma cells suppresses the osteoclast differentiation of bone marrow-derived macrophages (BMMs)

**Fig. 3:**
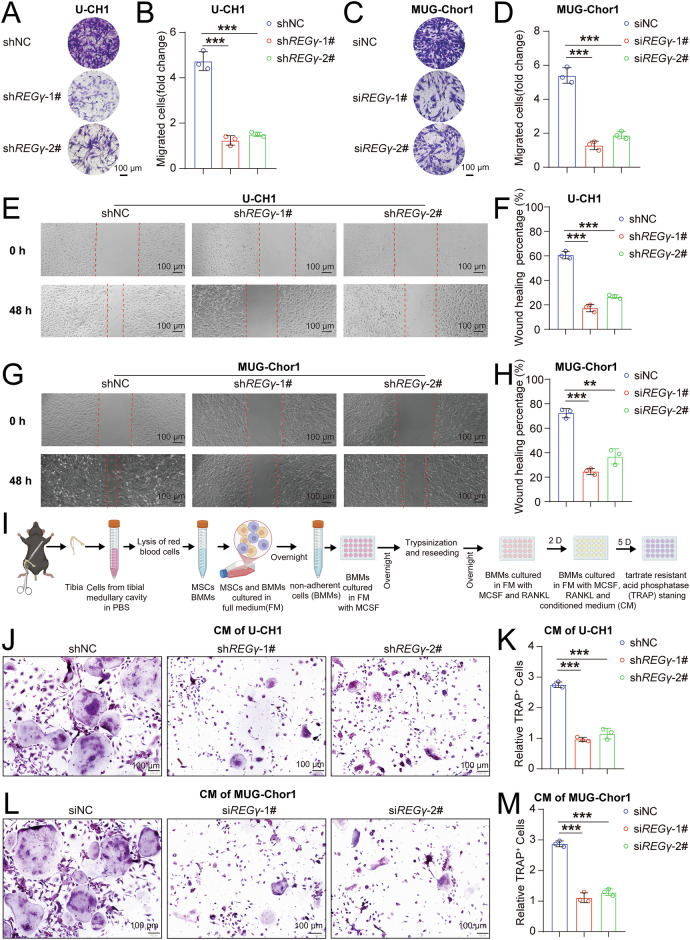
REGγ promotes the metastasis of chordoma cells, and conditioned medium from REGγ-inhibited chordoma cells suppresses the osteoclast differentiation of bone marrow-derived macrophages (BMMs). Transwell assays were used to assess migration in shNC, sh*REGγ*-1#, and sh*REGγ*-2# U-CH1 cells (**A**), with statistical results in (**B**). Transwell assays were used to assess migration in siNC, si*REGγ*-1#, and si*REGγ*-2# MUG-Chor1 cells (**C**), with statistical results in (**D**). Wound healing assays were used to evaluate migration in shNC, sh*REGγ*-1#, and sh*REGγ*-2# U-CH1 cells (**E**), with statistical results in (**F**). Wound healing assays were used to evaluate migration in siNC, si*REGγ*-1#, and si*REGγ*-2# MUG-Chor1 cells (**G**), with statistical results in (**H**). **I** Schematic of bone marrow-derived macrophages (BMMs) differentiation induced by conditioned medium (CM) from chordoma cells. Representative TRAP-stained images of BMMs treated with RANKL and MCSF, with CM from U-CH1 cells (**J**), with statistical results in (**K**). Representative TRAP-stained images of BMMs treated with RANKL and MCSF, with CM from MUG-Chor1 cells (**L**), with statistical results in (**M**). Scale bars: 100 µm.

Cell migration plays a key role in various physiological and pathological processes, including morphogenesis, wound healing, immune responses, and cancer invasion or metastasis [37]. Previous studies have demonstrated that REGγ knockdown inhibits migration and invasion in several cancers [38]. To further investigate the role of REGγ in chordoma cell migration, we conducted Transwell and wound healing assays. Transwell assay revealed that *REGγ* knockdown significantly reduced the migration in both U-CH1 and MUG-chor1 chordoma cells (Fig. 3A–D). Similarly, the wound healing assay confirmed that *REGγ* knockdown significantly inhibited the migration and invasion abilities of these cells (Fig. 3E–H). Bone tumor cells stimulate osteoclast activity, promoting bone resorption and causing bone destruction, fractures, and pain [39–42]. Osteosarcoma, a malignant bone cancer, is often characterized by bone loss due to increased osteoclast activity. Activated osteoclasts promote bone resorption and support osteosarcoma progression by secreting various cytokines [43]. This raises the question of whether chordoma, as a bone tumor, also causes bone damage during its development. To investigate this phenomenon, we added conditioned medium from chordoma cell culture to stimulate osteoclast differentiation of BMMs (Fig. 3I). After differentiation, TRAP staining was performed. The results showed that compared to conditioned medium from shNC chordoma cells, conditioned medium from sh*REGγ*-1# chordoma cells inhibited the differentiation of BMMs into osteoclasts (Fig. 3J–M), indicating that the ability of *REGγ*-knockdown cells to destroy bone was reduced.

### REGγ regulates the occurrence and development of chordoma via ubiquitin- and ATP-independent protein degradation of RIT1

**Fig. 4:**
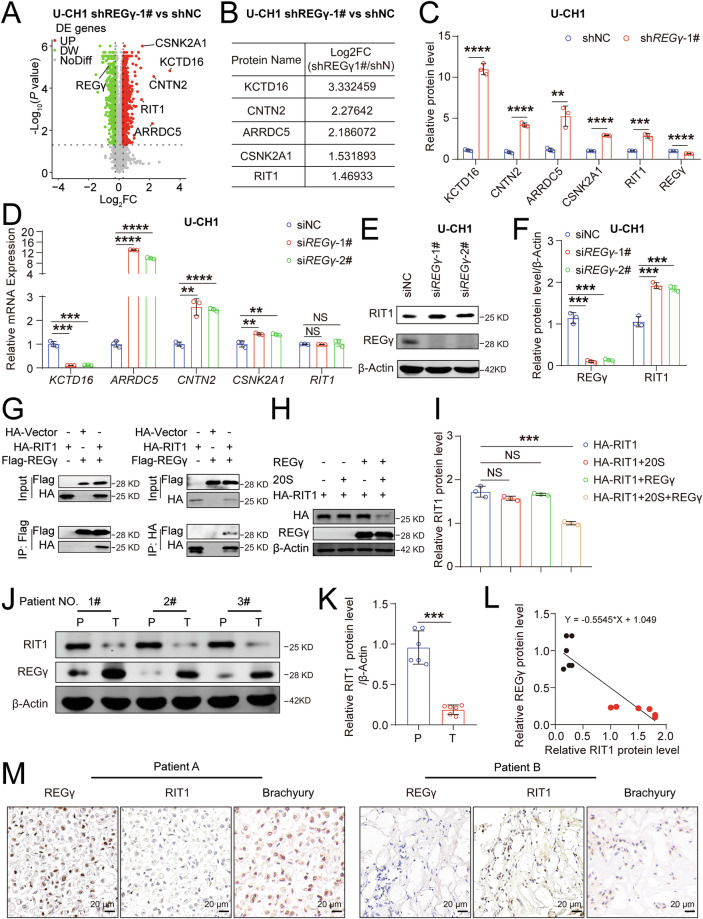
REGγ regulates the occurrence and development of chordoma via ubiquitin- and ATP-independent protein degradation of RIT1. **A** Differentially expressed proteins in shNC and sh*REGγ*-1# U-CH1 cells, as shown in volcano plots. **B** Top 5 upregulated proteins in sh*REGγ*-1# U-CH1 cells compared with shNC U-CH1 cells. **C** Relative protein levels of the top 5 upregulated proteins in sh*REGγ*-1# U-CH1 cells compared with shNC U-CH1 cells from mass spectrometry analysis. **D** qRT-PCR analysis of mRNA levels of the top 5 upregulated proteins, si*REGγ*-1# and si*REGγ*-2# U-CH1 cells compared with those in siNC U-CH1 cells. Western blot analysis of the protein levels of REGγ, RIT1, and β-Actin in siNC, si*REGγ*-1#, and si*REGγ*-2# U-CH1 cells (**E**), with statistical results in (**F**). **G** Co-IP analysis was used to assess the interaction between REGγ (Flag tag) and RIT1 (HA tag) in U-CH1 cells. Western blot analysis of REGγ, RIT1 (HA tag), and β-Actin after the degradation of RIT1 in vitro (**H**), with statistical results in (**I**). Western blot analysis of REGγ and RIT1 protein levels in chordoma (CHO) tissues and matched paracancerous (PARA) tissues (**J**), with statistical results in (**K**). **L** Correlation between RIT1 and REGγ protein expression in chordoma (CHO, black dot) tissues and matched paracancerous (PARA, red dot) tissues. **M** Representative IHC staining of REGγ, RIT1, and Brachyury expression in chordoma tissues. Scale bar = 20 µm.

REGγ, a member of the 11S proteasome activator family, degrades proteins in a ubiquitin and ATP-independent manner, promoting the degradation of key proteins such as SRC-3, p21, p53, p16, CK1δ, and SirT1, thus regulating various diseases [15–17, 19, 44–46]. REGγ is highly expressed in multiple cancers, including colon, lung, gastric, and renal cancers, and plays a critical role in tumorigenesis and progression [16, 22–24]. We have demonstrated that REGγ regulates the occurrence and development of chordoma, but the underlying mechanisms remain unclear. To investigate this, mass spectrometry analysis was conducted on sh*REGγ*-1# and shNC U-CH1 cells, revealing 1252 differentially expressed proteins, with 755 upregulated and 497 downregulated. The top five upregulated proteins in sh*REGγ*-1# U-CH1 cells compared with those in shNC cells were KCTD16, CNTN2, ARRDC5, CSNK2A1, and RIT1 (Fig. 4A–C). To explore the regulation of these proteins by REGγ, we performed quantitative PCR analysis. While the transcription levels of *KCTD16*, *CNTN2*, *ARRDC5*, *CSNK2A1*, and *RIT1* were measured, only *RIT1* showed no significant change in expression in *REGγ* knockdown UCH1 cells compared to control cells (Fig. 4D). Meanwhile, we have detected the expression of ARRDC5, CSNK2A1, and RIT1 (which were identified as differentially expressed proteins by mass spectrometry) and found that they were upregulated in cells with REGγ knockdown (Fig. S3C–D). Additionally, we examined the CPTAC database and found that RIT1 was significantly downregulated in breast cancer, kidney cancer, uterine corpus endometrial carcinoma, hepatocellular carcinoma, lung adenocarcinoma, lung squamous cell carcinoma, and head and neck squamous carcinoma (Fig. S3E–H). Inhibiting the expression of RIT1 was shown to promote the proliferation of chordoma cells and reduce apoptosis (Fig. S3L–S). Notably, transient knockdown of *REGγ* in chordoma cells resulted in a significant increase in RIT1 protein levels (Figs. 4E, F and S3A, B), suggesting that REGγ may regulate RIT1 via a ubiquitin- and ATP-independent mechanism. Co-immunoprecipitation assay confirmed that REGγ interacts with RIT1 (Fig. 4G), and the in vitro degradation assay further demonstrated that REGγ can degrade RIT1 through this ubiquitin- and ATP-independent pathway (Fig. 4H, I). Furthermore, RIT1 expression was significantly lower in chordoma tissue samples than in matched paracancerous (PARA) tissue samples, and RIT1 expression was negatively correlated with REGγ expression (Fig. 4J–M). Together, these findings might indicate that REGγ regulates chordoma progression by mediating the degradation of RIT1 through a ubiquitin- and ATP-independent pathway.

### RIT1 plays a crucial role in the cellular processes regulated by REGγ in chordoma progression

**Fig. 5:**
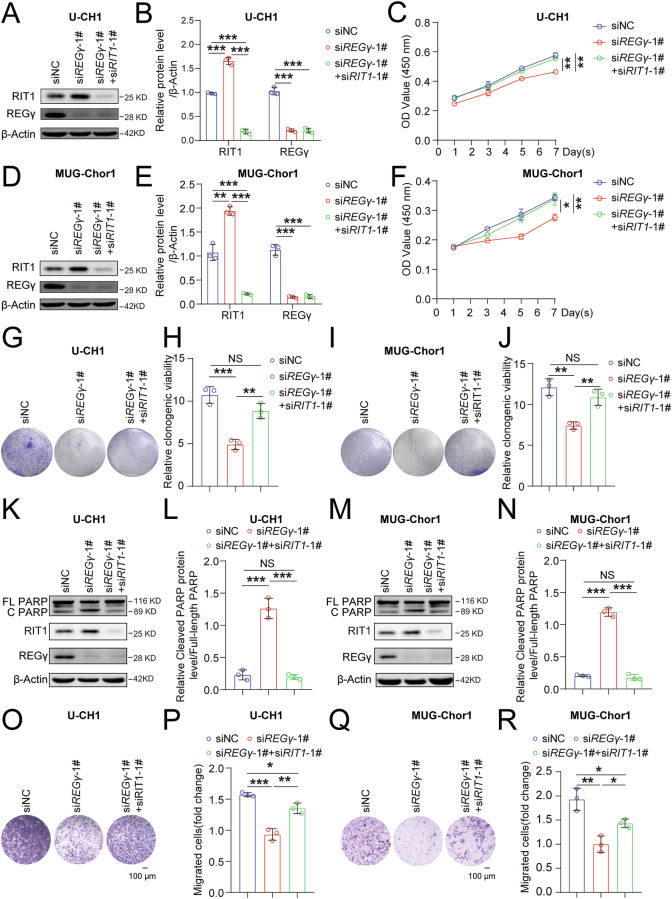
RIT1 plays a crucial role in the cellular processes regulated by REGγ in chordoma progression. Western blot analysis of REGγ, RIT1, and β-Actin expression in siNC, si*REGγ*-1#, and si*REGγ*-1#+si*RIT1*-1# U-CH1 cells (**A**), with statistical results in (**B**). **C** A CCK8 assay was used to evaluate the effect of REGγ on cell proliferation in siNC, si*REGγ*-1#, and si*REGγ*-1#+si*RIT1*-1# U-CH1 cells. Western blot analysis of REGγ, RIT1 and β-Actin expression in siNC, si*REGγ*-1#, and si*REGγ*-1#+si*RIT1*-1# MUG-Chor1 cells (**D**), with statistical analysis results in (**E**). **F** A CCK8 assay was used to evaluate the effect of REGγ on cell proliferation in siNC, si*REGγ*-1#, and si*REGγ*-1#+si*RIT1*-1# MUG-Chor1 cells. Colony formation ability in siNC, si*REGγ*-1#, and si*REGγ*-1#+si*RIT1*-1# U-CH1 cells (**G**), with statistical results in (**H**). Colony formation ability in siNC, si*REGγ*-1#, and si*REGγ*-1#+si*RIT1*-1# MUG-Chor1 cells (**I**), with statistical results in (**J**). Cell apoptosis was assessed by flow cytometry after Annexin V-APC staining in siNC, si*REGγ*-1#, and si*REGγ*-1#+si*RIT1*-1# U-CH1 cells (**K**), with the ratio of cleaved PARP to full-length PARP (**L**). Cell apoptosis was evaluated by flow cytometry after Annexin V-APC staining in siNC, si*REGγ*-1#, and si*REGγ*-1#+si*RIT1*-1# MUG-Chor1 cells (**M**), with the ratio of cleaved PARP to full-length PARP (**N**). Transwell assays were used to assess migration and invasion in siNC, si*REGγ*-1#, and si*REGγ*-1#+si*RIT1*-1# U-CH1 cells (**O**), with statistical results in (**P**). Transwell assays were used to assess migration and invasion in siNC, si*REGγ*-1#, and si*REGγ*-1#+si*RIT1*-1# MUG-Chor1 cells (**Q**), with statistical results in (**R**). FL PARP: full-length PARP C PARP: cleaved PARP.

To investigate the role of RIT1 in REGγ-mediated chordoma progression, we first knocked down REGγ, RIT1, or both in U-CH1 cells (Fig. 5A, B). The results of the CCK8 assays revealed that double-knockdown of REGγ and RIT1 rescued the decreased cell proliferation caused by *REGγ* knockdown in U-CH1 cells (Fig. 5C) and MUG-Chor1 cells (Fig. 5D–F). Colony formation assays confirmed that inhibiting RIT1 expression alleviated the reduction in proliferation resulting from *REGγ* knockdown in both U-CH1 and MUG-Chor1 cells (Fig. 5G–J). Western blot analysis revealed that inhibiting RIT1 expression reduced the increased apoptosis induced by *REGγ* knockdown in both U-CH1 and MUG-Chor1 cells (Fig. 5K–N). Transwell assays revealed that inhibiting RIT1 expression also mitigated the migration decrease caused by *REGγ* knockdown in both U-CH1 and MUG-Chor1 cells (Fig. 5O–R). Additionally, an experiment using conditioned medium from chordoma cells was used to stimulate BMMs to differentiate into osteoclasts, revealing that conditioned medium from REGγ and RIT1 double-knockdown chordoma cells mitigated the reduction in osteoclast differentiation induced by *REGγ* knockdown in both U-CH1 and MUG-Chor1 cells (Fig. S4A–D). These findings suggest that RIT1 plays a crucial role in the cellular processes regulated by REGγ in chordoma progression.

### REGγ regulates chordoma through regulating the RIT1-MAPK pathway

**Fig. 6:**
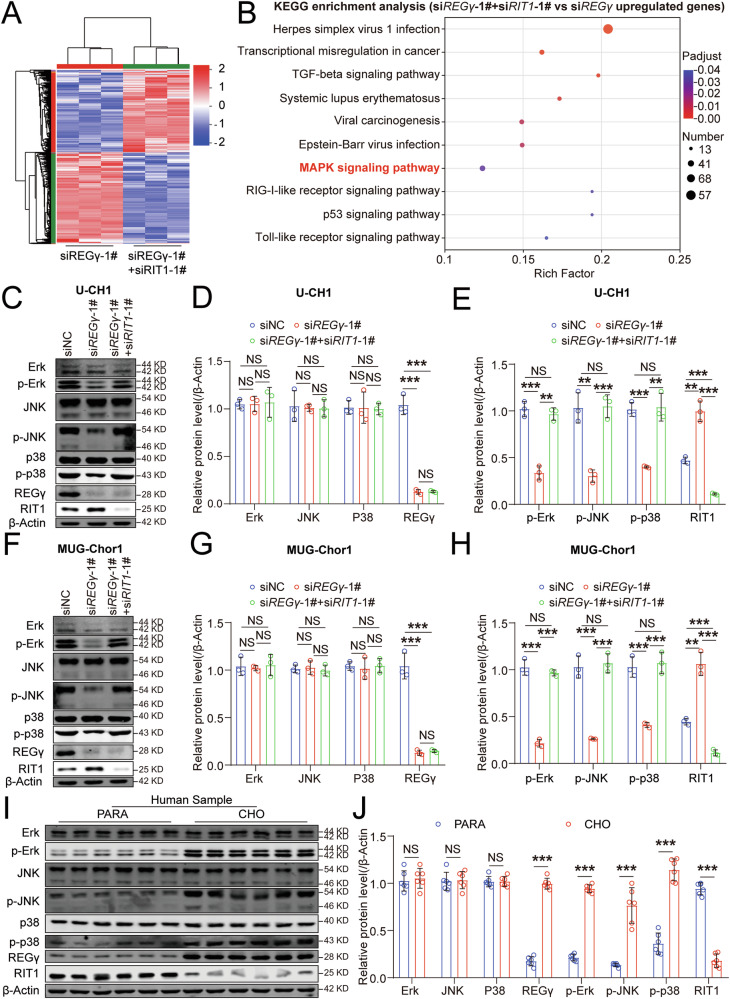
REGγ regulates chordoma through regulating the RIT1-MAPK pathway. **A** Heatmap showing differentially expressed genes in si*REGγ*-1#+si*RIT1*-1# cells compared with si*REGγ*-1# U-CH1 cells by RNA-seq. **B** KEGG analysis of upregulated gene in si*REGγ*-1#+si*RIT1*-1# cells compared with si*REGγ*-1# cells. Western blot analysis of REGγ, RIT1, Erk, JNK p38, p-Erk, p-JNK p-p38, and β-Actin expression in siNC, si*REGγ*-1#, and si*REGγ*-1#+si*RIT1*-1# U-CH1 cells (**C**), with statistical results in (**D**, **E**). Western blot analysis of REGγ, RIT1, Erk, JNK p38, p-Erk, p-JNK p-p38, and β-Actin expression in siNC, siREGγ-1#, and si*REGγ*-1#+si*RIT1*-1# MUG-Chor1 cells (**F**), with statistical results in (**G**, **H**). Western blot analysis of REGγ, RIT1, Erk, JNK, p38, p-Erk, p-JNK, p-p38, and β-Actin expression in chordoma (CHO) tissues and matched paracancerous (PARA) tissues (**I**), with statistical results in (**J**) (*n* = 6).

To explore the molecular mechanisms of RIT1 in REGγ-mediated chordoma progression, mass spectrometry analysis was conducted on U-CH1 cells with double-knockdown of *REGγ* and *RIT1*, as well as those with *REGγ* knockdown alone. This analysis revealed 3992 differentially expressed proteins, with 1819 upregulated and 2173 downregulated (Fig. 6A). KEGG pathway analysis revealed that the upregulated proteins in the double-knockdown group were strongly associated with the MAPK pathway compared to the R*EGγ* knockdown group (Fig. 6B). We further used western blotting to assess the expression of key MAPK pathway proteins—Erk, JNK, p38, p-Erk, p-JNK, and p-p38—in U-CH1 cells with a negative control, double knockdown of *REGγ* and *RIT1*, as well as *REGγ* knockdown alone. The results revealed that p-Erk, p-JNK, and p-p38 levels were reduced in *REGγ* knockdown cells than that in control cells, but double knockdown of *REGγ* and *RIT1* mitigated this reduction (Fig. 6C–E). These findings were further validated in MUG-Chor1 cells (Fig. 6F–H). Furthermore, we examined the expression of REGγ, RIT1, and MAPK pathway proteins in chordoma and matched paracancerous tissues. We observed significant upregulation of REGγ and MAPK proteins (p-Erk, p-JNK, and p-p38) in chordoma tissues, whereas RIT1 was downregulated (Fig. 6I, J). This observation aligns with the cellular data, suggesting that REGγ regulates chordoma progression through the RIT1-MAPK pathway.

### REGγ regulates chordoma through the RIT1-MAPK pathway at the patient-derived organoid (PDO) level

**Fig. 7:**
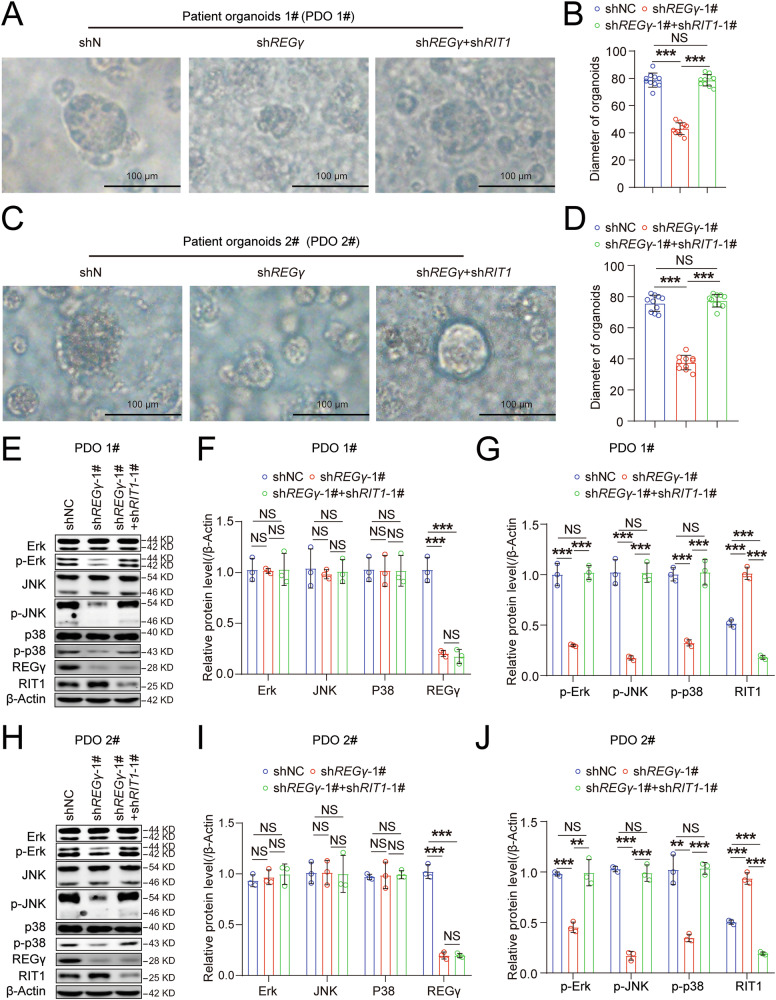
REGγ regulates chordoma through the RIT1-MAPK pathway at the patient-derived organoid (PDO) level. Representative images of patient 1#-derived chordoma organoids treated with shNC, shREGγ, and sh*REGγ*+sh*RIT1* (**A**), with statistical results in (**B**). Representative images of patient 2#-derived chordoma organoids treated with shNC, sh*REGγ*, and sh*REGγ*+sh*RIT1* (**C**), with statistical results in (**D**). Western blot analysis of REGγ, RIT1, Erk, JNK, p38, p-Erk, p-JNK, p-p38, and β-Actin expression in patient 1#-derived chordoma organoids treated with shNC, sh*REGγ*, and sh*REGγ*+sh*RIT1* (**E**), with statistical results in (**F**, **G**). Western blot analysis of REGγ, RIT1, Erk, JNK, p38, p-Erk, p-JNK, p-p38, and β-Actin expression in patient 1#-derived chordoma organoids treated with shNC, sh*REGγ*, and sh*REGγ*+sh*RIT1* (**H**), with statistical results in (**I**, **J**).

**Fig. 8:**
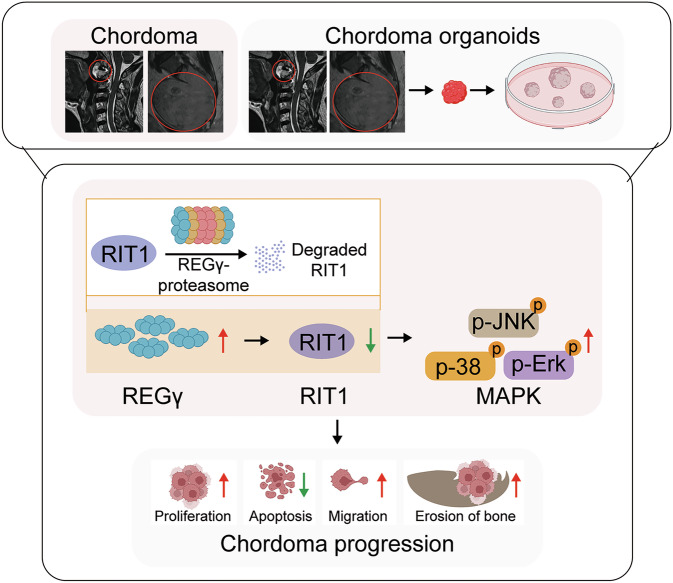
REGγ regulates the RIT1-MAPK pathway in chordoma progression. REGγ regulates chordoma progression through the ubiquitin- and ATP-independent degradation of RIT1, which modulates the RIT1-MAPK pathway.

Patient-derived organoids are powerful tools in cancer research, offering models that replicate the structure and diversity of primary tumors [47]. These models preserve key histological and molecular characteristics, aiding the study of tumor biology, molecular pathways, and the tumor immune environment [48]. Therefore, we cultured chordoma PDOs (Fig. S5A) and validated the mechanism of REGγ regulation in chordoma at the PDO level. We found that knockdown of REGγ inhibited the growth of chordoma PDOs and promoted their apoptosis. However, the double knockdown of REGγ and RIT1 alleviated both the slow growth and the high apoptosis of chordoma PDOs caused by REGγ knockdown (Figs. 7A–D and S5B–E). Additionally, an experiment using conditioned medium from chordoma PDOs was used to stimulate BMMs to differentiate into osteoclasts, revealing that conditioned medium from *REGγ* and *RIT1* double-knockdown chordoma PDOs mitigated the reduction in osteoclast differentiation induced by *REGγ* knockdown (Fig. S5B–E). Mechanistically, we found that p-Erk, p-JNK, and p-p38 levels were reduced in *REGγ* knockdown PDOs than in control PDOs, but double knockdown of *REGγ* and *RIT1* mitigated this reduction (Fig. 7E–J). These findings emphasize the importance of REGγ in regulating chordoma growth through the RIT1-MAPK pathway (Fig. 8).

## Discussion

In this study, we found that REGγ expression was significantly upregulated in chordoma tissues compared with paracancerous tissues, and its high expression was closely associated with poor prognosis. Additionally, we demonstrated that reduced REGγ expression inhibited chordoma cell proliferation and colony formation, induced apoptosis, and suppressed their bone-destructive capacity. Mechanistically, REGγ regulates the degradation of RIT1 through a ubiquitin- and ATP-independent pathway. Further analysis revealed that REGγ promotes chordoma progression by modulating the RIT1-MAPK signaling pathway. Organoid-based experiments further validated the in vitro findings. In summary, REGγ is a novel biomarker for chordoma and holds significant potential for targeted therapy.

The proteasome system is one of the key regulatory systems in the human body, and is responsible for intracellular protein degradation, accounting for over 80% of cellular protein turnover. It is crucial for maintaining normal cell function [11]. Disruption of this system can lead to metabolic disturbances in cells, potentially triggering diseases such as inflammation and cancer [12]. The proteasomal degradation system is classified into ATP-ubiquitin-dependent and ATP-ubiquitin-independent pathways [13]. The Proteasome activator REGγ, a member of the REG (11S) family, was first identified as the Ki antigen in autoantibodies from the serum of patients with systemic lupus erythematosus. REGγ functions as a proteasome activator, promoting protein degradation through a ubiquitin- and ATP-independent mechanism [15]. Studies have shown that REGγ enhances the degradation of key target proteins, such as SRC-3, p21, p53, p16, CK1δ, and SirT1, thereby regulating the progression of various diseases [16, 17, 19, 44–46]. Furthermore, REGγ is highly expressed in several types of cancer, including colon, lung, gastric, and kidney cancers, and plays a significant role in tumorigenesis and progression [16, 22–24]. While the role of REGγ in cancer development has gained increasing attention as a new avenue of cancer research, its involvement in the onset and progression of chordoma remains unexplored and requires further investigation. Our study reveals the regulatory mechanisms of REGγ in chordoma. We identified the biological functions of REGγ in regulating cell proliferation, apoptosis, migration, and bone destruction in chordoma cells. These findings align with the analysis of REGγ expression in chordoma patients, suggesting that the overexpression of REGγ may promote chordoma progression by increasing cell proliferation, inhibiting apoptosis, facilitating cell migration, and increasing bone destruction in chordoma cells.

To investigate the molecular mechanisms by which REGγ regulates chordoma, mass spectrometry analysis was conducted on sh*REGγ*-1# and shNC U-CH1 cells. Among the top five upregulated proteins in REGγ-knockdown chordoma cells, only the transcription level of RIT1 remained unchanged. RIT1, a small GTPase related to RAS with structural similarities to KRAS, alternates between GDP- and GTP-bound forms, activating downstream MAPK and AKT pathways when bound to GTP [30, 31, 34, 49]. Although RIT1 modulates MAPK signaling, its regulation varies across cell types [35, 49, 50]. We identified RIT1 as a key protein influenced by REGγ, and further experiments confirmed that REGγ interacts with and degrades RIT1, which is downregulated in chordoma tissues. Thus, we further demonstrated that RIT1 plays a crucial role in the cellular processes regulated by REGγ in chordoma progression.

To explore the molecular mechanisms by which RIT1 contributes to REGγ-driven chordoma progression, mass spectrometry analysis was performed on U-CH1 cells with either double knockdown of *REGγ* and *RIT1* or *REGγ* knockdown alone. KEGG pathway analysis revealed that the upregulated proteins in the double-knockdown group compared with the *REGγ*-knockdown group were closely linked to the MAPK pathway. Various extracellular signals activate the MAPK pathway through Ras/Raf, which phosphorylates MAPK. Phosphorylated MAPK then enters the nucleus, where it participates in various physiological processes such as cell growth, development, proliferation, and differentiation [51, 52]. Abnormal activation or overactivation of the MAPK signaling pathway plays a critical role in cell malignant transformation and progression [51]. Studies have shown that MAPKs are closely associated with the development and progression of cancers, including breast, ovarian, esophageal, colon, gastric, and liver cancer [53–62]. In summary, we hypothesize that REGγ promotes chordoma progression by regulating the RIT1-MAPK pathway. We found that p-Erk, p-JNK, and p-p38 levels were reduced in *REGγ* knockdown chordoma cells and PDOs than in controls, but double knockdown of *REGγ* and *RIT1* mitigated this reduction. These findings reveal that REGγ regulates the RIT1-MAPK pathway in chordoma progression and provide new insights for targeted therapy of chordoma.

## Supplementary information

Supplementary information

Uncropped original western blots

## Data Availability

Raw data are available from the corresponding authors on reasonable request.
