# Supplementary material for: Ubiquitin-independent pathway regulates the RIT1-MAPK pathway in chordoma progression
Source: Cell Death Dis. 2025 Oct 24;16(1):756. doi: 10.1038/s41419-025-08092-z (PMC12552625; doi:10.1038/s41419-025-08092-z)
Supplement: Supplementary file 1 — Supplementary information [file 41419_2025_8092_MOESM1_ESM.pdf]

Supplementary file

SUPPLEMENTARY METHODS

Mass spectrometry analysis

Protein extraction was performed using SDS lysis buffer containing protease inhibitors, followed by BCA quantification. Then, 100 μg of protein was reduced, alkylated, acetone-precipitated, trypsin-digested, and labeled with TMT reagents.The peptide samples were re-solubilized with UPLC loading buffer (2% acetonitrile (ammonia to pH 10)) and separated in high pH liquid phase using a reversed-phase C18 column ACQUITY UPLC BEH C18 Column. LC-MS/MS analysis Two-dimensional analysis was performed by liquid chromatography tandem mass spectrometry (Evosep One combined with Obitrap Exploris 480 mass spectrometer) according to the standard protocols by Majorbio Bio-Pharm Technology Co. Ltd. (Shanghai, China). And the thresholds of fold change (>1.2 or <0.83) and P-value <0.05 were used to identify differentially expressed proteins (DEPs).

RNA-seq analysis

Total RNA was extracted from the tissue using TRIzol® Reagent according to the manufacturer's instructions. Using 1 μg of total RNA, mRNA was enriched via oligo(dT) beads, fragmented, and converted to double-stranded cDNA to construct strand-specific libraries through end repair, phosphorylation, and A-tailing. The libraries were size-selected for 300 bp fragments using 2% agarose gel electrophoresis, amplified with Phusion DNA polymerase for 15 PCR cycles, and quantified by Qubit 4.0. Paired-end RNA sequencing was performed on the Illumina NovaSeq Xplus platform. Reads containing adapter contamination, low-quality bases, and undetermined bases with default parameters were removed using Fastp software (https://github.com/OpenGene/fastp). Sequence quality was further verified using Fastp. HiSat2 software was used to align the quality-controlled raw data with the reference genome to obtain mapped reads for subsequent transcript assembly, expression calculation. Gene and transcript expression levels were quantified using RSEM software, and differentially expressed genes were identified using DESeq2 or DEGseq software. And the p-value <0.05 and |fold change| ≥2 was used to identify differential genes. GO enrichment analysis was performed by clusterProfiler,

with a p-value <0.05 considered to be a significant enrichment.

In vitro protein degradation assay

The in vitro proteolytic system includes 5 μL RIT1 protein, 0.25 μg 20S proteasome, and 2 μg REGγ heptameric, with a total reaction volume of 50 μL. The RIT1 protein was translated using the TNT® T7 Quick Coupled Transcription/Translation System (Promega). The reaction system was incubated at 30 °C for 4 hours, and the results were analyzed by Western blotting.

Wound healing assay

After counting the successfully transfected cells, seed 1×10^5 cells per well into 12-well plates and reach 100% confluence. Scratch the wound on the monolayer of cells with a 10 μL pipette tip. The cells were then cultured at 37°C and 5% CO_2 and images were captured at 0, 48 h. Each experiment was repeated three times.

In vitro osteoclast differentiation assay

Bone marrow cells were isolated from the tibia of 6-8-week-old mice and cultured overnight using α-MEM medium containing 10% fetal bovine serum (FBS). Transfer the unadherent cells to a new plate containing 10 ng/mL macrophage colony-stimulating factor (M-CSF) for continued overnight culture. Bone marrow-derived macrophages (BMMs) were then digested and resuspended at a density of 1×10^5/mL in the same medium for further culture, and replaced with osteoclast differentiation medium (α-MEM 10% FBS 10 ng/mL M-CSF 50 ng/mL RANKL) the next day. Two days later, the differentiation medium prepared with the collected conditioned medium was changed, and three days later TRAP staining was performed.

Construction of a tissue microarray (TMA) followed by immunohistochemical (IHC) staining

The tissue microarray (TMA) was constructed from 171 individual conventional chordoma patient specimens within a 4% formalin‑fixed and then paraffin‑embedded (FFPE) block, using 1.5mm diameter cores. All samples were collected from the archives of the Spine Tumor Center, Chanzheng Hospital. The chips were baked on a heating plate at 62°C for 2 hours, followed by

sequential dewaxing and rehydration. Antigen retrieval was performed in EDTA buffer at 100°C for 20 min, then cooled to room temperature. Endogenous peroxidase activity was blocked with 3% H_2O_2 for 10 min, followed by blocking with 5 % BSA at 37 °C for 30 min. Sections were incubated with primary antibody overnight at 4 °C, then with secondary antibody at 37 °C for 30 min. Then DAB chromogenic agent was added dropwise, and the color development effect was observed under the microscope. Hematoxylin staining was then performed, subjected to graded dehydration, and then mounted with a drop of neutral resin and dried for photography.

Patient-Derived Organoid (PDO) Establishment and Culture

Tumors are obtained by surgical resection and processed within 24 hours for organoid culture. Cut the tumor tissue into small pieces and put it in a 15ml centrifuge tube, add 4-5ml of digestive solution (DB) for digestion, digest the tumor tissue on a 37°C shaker for 30 minutes, filter the cell suspension with a strainer, centrifuge the cell suspension with 1ml of split red solution, resuspend the cells on ice for 1-2 min and then centrifuge, then add 1ml of washing solution to resuspend the cells, and use AO/PI staining method for cell counting. Resuspend cells (10^4/10ul) in a 48-well plate using Matrigel, place in a 37°C incubator, and let stand for 10-15 minutes to add (AEBL) medium. After 24 hours, shREGγ/shREGγ-shRIT1 virus was added dropwise and the medium was changed every 2-3 days.

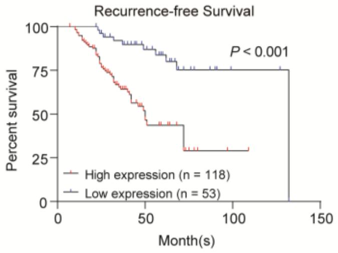

Fig. S1 REGγ is upregulated in chordoma and correlates with poor prognosis. Kaplan-Meier curves of recurrence-free survival (RFS) based on REGγ expression in a conventional chordoma tissue microarray.

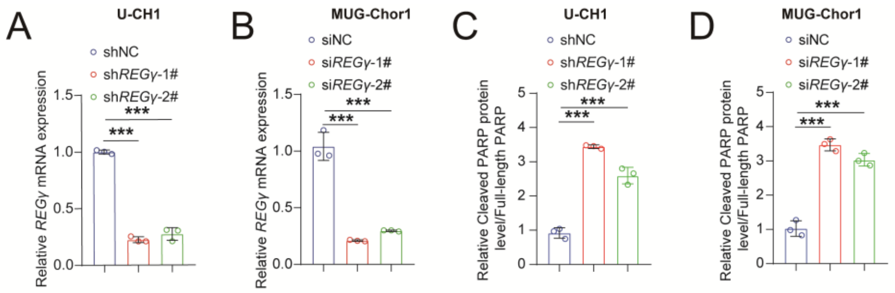

Fig. S2 REGγ inhibits apoptosis of chordoma cells**. A** qRT-PCR assay measured *REGγ* expression in shNC, sh*REGγ*-1#, and sh*REGγ*-2# U-CH1 cells. **B** qRT-PCR assay measured *REGγ* expression in siNC, si*REGγ*-1#, and si*REGγ*-2# MUG-Chor1 cells. **C** The ratio of cleaved PARP to full-length PARP of Fig 2O. **D** The ratio of cleaved PARP to full-length PARP of Fig 2P. FL PARP: full-length PARP C PARP: cleaved PARP.

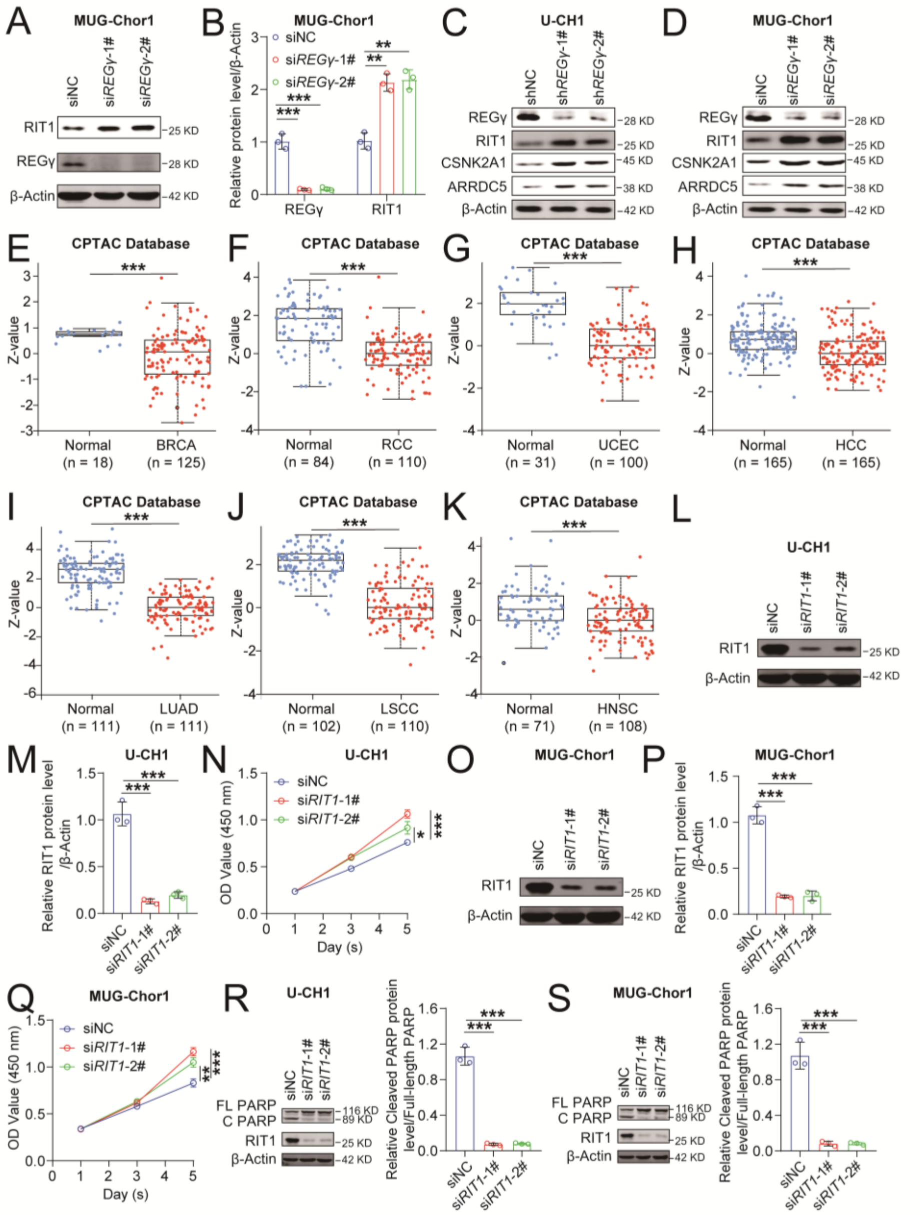

Fig. S3 The protein level of RIT1 is lowly expressed in many cancers and Inhibition of RIT1 expression in chordoma cells promotes cell proliferation. **A-B** Western blot analysis of REGγ, RIT1 and β-Actin expression in siNC, si*REGγ*-1#, and si*REGγ-2#* MUG-Chor1 cells (A), with statistical results (B). **C** Western blot analysis of REGγ, RIT1, CSNK2A1, ARRDC5 and β-Actin expression in shNC, sh*REGγ*-1#, and sh*REGγ*-2# U-CH1 cells. **D** Western blot analysis of REGγ, RIT1, CSNK2A1, ARRDC5 and β-Actin expression in siNC, si*REGγ*-1#, and si*REGγ*-2# MUG-Chor1 cells. **E** RIT1 protein expression in normal and breast cancer (BRCA) tissues from CPTAC database. **F** RIT1 protein expression in normal and renal cell carcinoma (RCC) tissues in CPTAC

database. **G** RIT1 protein expression in normal and uterine corpus endometrial carcinoma (UCEC) tissues in CPTAC database. **H** RIT1 protein expression in normal and hepatocellular carcinoma (HCC) tissues in CPTAC database. **I** RIT1 protein expression in normal and lung adenocarcinoma (LUAD) tissues in CPTAC database. **J** RIT1 protein expression in normal and lung squamous cell carcinoma (LSCC) tissues in CPTAC database. **K** RIT1 protein expression in normal and head and neck squamous carcinoma (HNSC) tissues in CPTAC database. **L-M** Western blot analysis of RIT1 and β-Actin expression in siNC, si*RIT1*-1#, and si*RIT1*-2# U-CH1 cells (L), with statistical results (M). **N** A CCK8 assay was used to evaluate the effect of RIT1 on cell proliferation in siNC, si*RIT1*-1#, and si*RIT1*-2# U-CH1 cells. **O-P** Western blot analysis of RIT1 and β-Actin expression in siNC, si*RIT1*-1#, and si*RIT1*-2# MUG-Chor1 cells (O), with statistical results (P). **Q** A CCK8 assay was used to evaluate the effect of RIT1 on cell proliferation in siNC, si*RIT1*-1#, and si*RIT1*-2# MUG-Chor1 cells. **R** Cell apoptosis was evaluated by WB analysis (the protein levels of cleaved PARP) in siNC, si*RIT1*-1#, and sh*RIT1*-2# U-CH1 cells, with the ratio of cleaved PARP to full-length PARP. **S** Cell apoptosis was evaluated by WB analysis (the protein levels of cleaved PARP) in siNC, si*RIT1*-1#, and si*RIT1*-2# MUG-Chor1 cells, with the ratio of cleaved PARP to full-length PARP. FL PARP: full-length PARP C PARP: cleaved PARP.

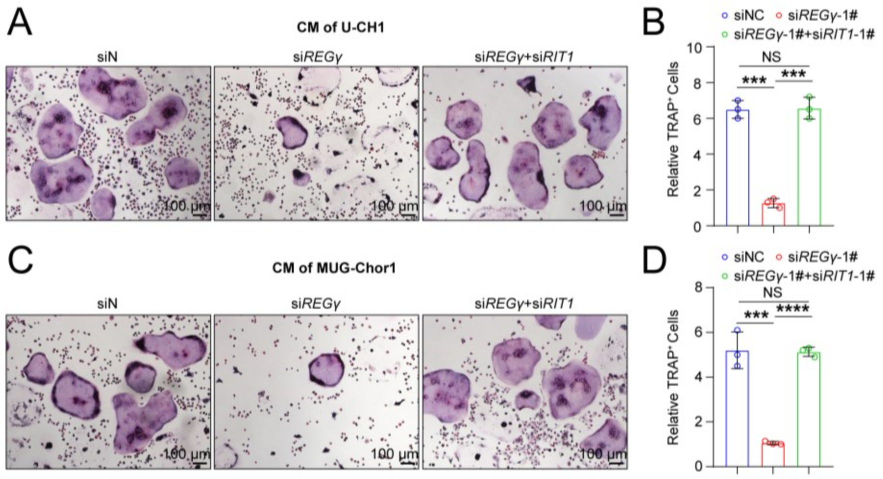

Fig. S4 The conditioned medium from REGγ and RIT1 double-knockdown can rescue the inhibition of osteoclast differentiation in BMM cells treated with REGγ knockdown conditioned medium. **A-B** Representative TRAP-stained images of BMMs treated with RANKL and MCSF, with CM from U-CH1 cells (A), and statistical results in (B). **C-D** Representative TRAP-stained images of BMMs treated with RANKL and MCSF, with CM from MUG-Chor1 cells (C), and statistical results in (D).

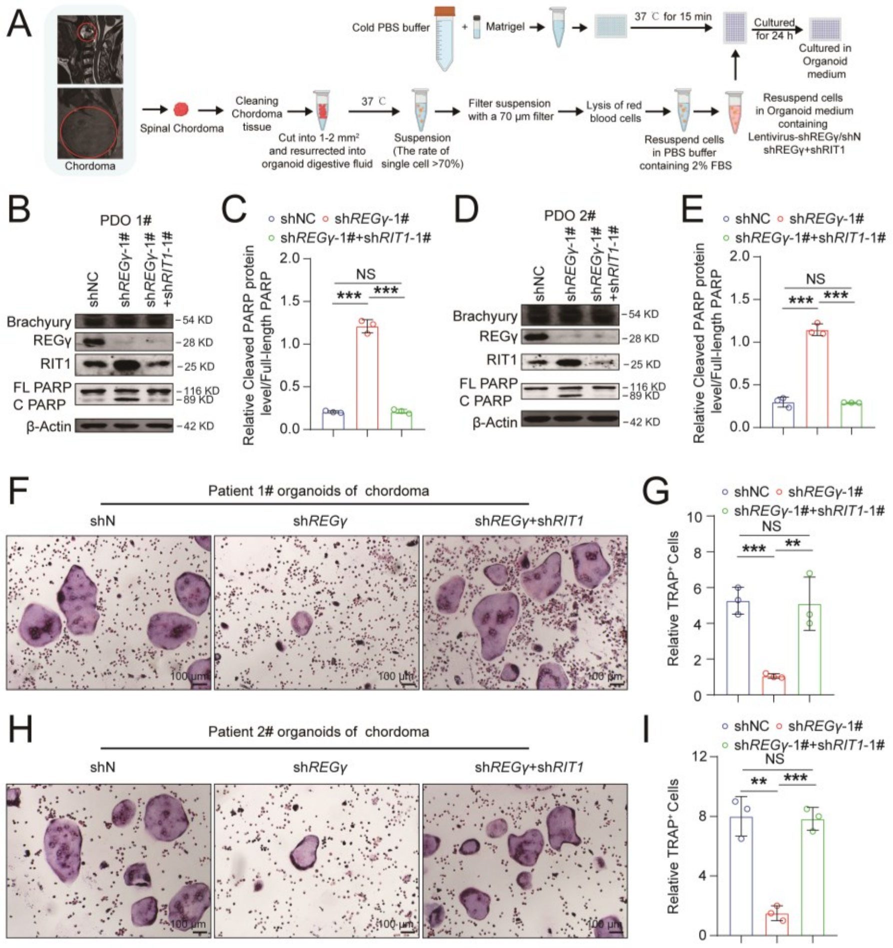

Fig. S5 REGγ regulates chordoma through the RIT1-MAPK pathway at the patient-derived organoid (PDO) level. **A** Flowchart of chordoma organoid construction. **B-C** Western blot analysis of REGγ, RIT1, Brachyury, Full-Length PARP, Cleaved-PARP and β-Actin expression in shNC, sh*REGγ*-1#, and sh*REGγ*-1#+sh*RIT1*-1# PDO1# cells(B), with the ratio of cleaved PARP to full-length PARP (C). **D-E** Western blot analysis of REGγ, RIT1, Brachyury, Full-Length PARP, Cleaved PARP and β-Actin expression in shNC, sh*REGγ*-1#, and sh*REGγ*-1#+sh*RIT1*-1# PDO2# cells(D), with the ratio of cleaved PARP to full-length PARP (E). **F-G** Representative TRAP-stained images of BMMs treated with RANKL and MCSF, with CM from patient 1# organoids of chordoma (F), and statistical results in (G). **H-I** Representative TRAP-stained images of BMMs treated with RANKL and MCSF, with CM from patient 2# organoids of chordoma (H), and statistical results in (I). FL PARP: full-length PARP C PARP: cleaved PARP.

Table S1 Primers and sequences

Primers for qPCR		
Gene	Forward primer sequence	Reverse primer sequence
hKCTD16	GCCTGTAACTCATCGGTGACAG	AGCAATCGCAGTGTGAGGGTGA
hARRDC5	CGTCGAATGGAGTGAAGAAGCC	GAAGTCAAAGGTGTGGCTGCCT
hCNTN2	TACGAGTGTGAGGCGGAGAACT	CAACGCAGGTTGGAGCCAATGT
hCSNK2A1	GGTGAGGATAGCCAAGGTTCTG	TCACTGTGGACAAAGCGTTCCC
hRIT1	CGTACTGACGATACACCTGTGG	GTAGCGGTATGCAGCAGATGTC
hREGγ	AAGGTTGATTCTTTCAGGGAGC	AGTGGATCTGAGTTAGGTCATG
Primers for Plasmid Construction		
Plasmid	Forward primer sequence	Reverse primer sequence
pSG5-HA-*RIT1*	ATACGGAATTCCGATGGTCATG GAAGTGGGCAC	GAGCGGGATCCCGTCAGCAGCT GGGAGAGGTCG
siRNA sequences		
Name	Sense sequence	Antisense sequence
si*RIT1*-1#(h)	CACGUCGAAGUUUCCAUGAAGU TT	ACUUCAUGGAAACUUCGACGUG TT
si*RIT1*-2#(h)	CGAGAAUUCAGCUGUCCCUUTT	AAGGGACAGCUGAAUUCUCGTT
si*REGγ*-1#(h)	CAGAAGACUUGGUGGCAAATT	UUUGCCACCAAGUCUUCUGTT
si*REGγ*-2#(h)	AGAAGUUAUUAGAACUUGAUA	UCAAGUUCUAAUAACUUCUUU
Oligos for shRNA Plasmid Construction		
pLVX-U6-sh*RIT1*-1#-CMV-mRFP1-T2A-Puro	ccggCACGTCGAAGTTTCCATGAA GTctcgagACTTCATGGAAACTTC GACGTGttttt	aattcaaaaaCACGTCGAAGTTTCCA TGAAGTctcgagACTTCATGGAAAC TTCGACGTG
pLVX-U6-sh*REGγ*-1#-gcGFP-IRES-Puro	ccggCAGAAGACTTGGTGGCAAA TTctcgagAATTTGCCACCAAGTCT TCTGttttt	aattcaaaaaCAGAAGACTTGGTGGC AAATTctcgagAATTTGCCACCAAG TCTTCTG
pLVX-U6-sh*REGγ*-2#-gcGFP-IRES-Puro	ccggAGAAGTTATTAGAACTTGAT ActcgagTATCAAGTTCTAATAACT TCTttttt	aattcaaaaaAGAAGTTATTAGAACTT GATActcgagTATCAAGTTCTAATAA CTTCT

Table S2. Patient characteristics and the analysis of potential relationship between the REGγ expression level and other clinical parameters

**Factor**		**n**	**Low expression**	**High expression**	**χ2**	**P value**
Age	<60, y	77	20	57	1.651	0.245
	≥60, y	94	33	61		
Primary/ Recurrent lesion	Primary lesion	107	38	69	2.731	0.098
	Recurrent lesion	64	15	49		
Gender	Male	103	37	76	0.477	0.601
	Female	68	16	42		
Surgical mode	En bloc resection	109	32	77	0.376	0.607
	Piecemeal resection	62	21	41		
Tumor size	≤6 cm	75	26	49	0.842	0.406
	>6 cm	96	27	69		
Intraoperative bleeding	≤2000 mL	126	40	86	0.127	0.851
	>2000 mL	45	13	32		
Karnofsky performance status (KPS) score	<60	35	11	24	0.617	0.735
	60-80	78	22	56		
	>80	58	20	38		
Lesion site	Sacral	100	35	65	2.546	0.281
	Thoracolumbar	13	2	11		
	Cervical	58	16	42		

Table S3. Univariate and multivariate analysis of the prognostic factors correlating recurrence free survival

**Factor**	**Univariate analysis**	**Multivariate analysis**		
	P value	95%CI	Hazard ration	P value
REG expression level, high/low	<0.001***	3.264-10.204	5.771	<0.001**
Primary/ Recurrent lesion	0.063*	0.391-1.278	1.042	0.839
Age, <60/≥60, y	0.149			
Gender, M/F	0.199			
Surgical mode, piecemeal resection/ en bloc resection	<0.001**	1.474-3.699	2.335	<0.001**
Tumor size, ≤6 cm/>6 cm	0.099*	0.391-1.275	0.706	0.249
Intraoperative bleeding,≤2000 ml/>2000 ml	0.547			
KPS score, <60/60-80/>80	0.138			
Lesion site, sacral/ thoracolumbar/ cervical spine	0.027**	0.676-1.289	0.933	0.676

Table S4. Univariate and multivariate analysis of the prognostic factors correlating overall survival

**Factor**	**Univariate analysis**	**Multivariate analysis**		
	P value	95%CI	Hazard ration	P value
REG expression level, high/low	<0.001***	1.744-7.307	3.569	<0.001***
Primary/ Recurrent lesion	0.403			
Age, <60/≥60, y	0.675			
Gender, M/F	0.225			
Surgical mode, piecemeal resection/ en bloc resection	0.001**	1.482-4.953	2.710	0.001**
Tumor size, ≤6 cm/>6 cm	0.601			
Intraoperative bleeding, ≤2000 ml/>2000 ml	0.189			
KPS score, <60/60-80/>80	0.345			
Lesion site, sacral/ thoracolumbar/ cervical spine	0.861			
