# Supplementary figures and images for: Ubiquitin-independent pathway regulates the RIT1-MAPK pathway in chordoma progression
Source: Cell Death Dis. 2025 Oct 24;16(1):756. doi: 10.1038/s41419-025-08092-z (PMC12552625; doi:10.1038/s41419-025-08092-z)

Fig. 1D

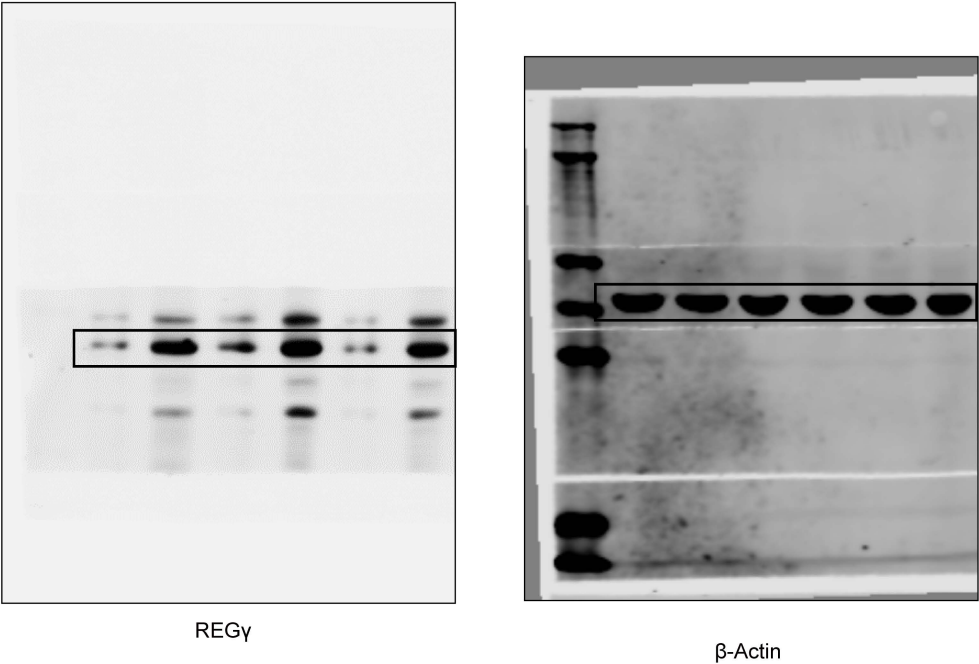

Fig. 2A

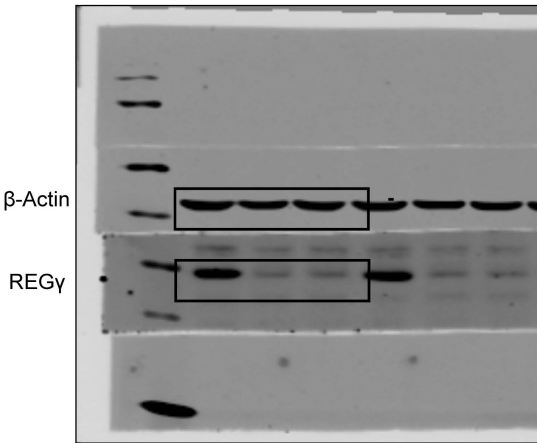

Fig. 2E

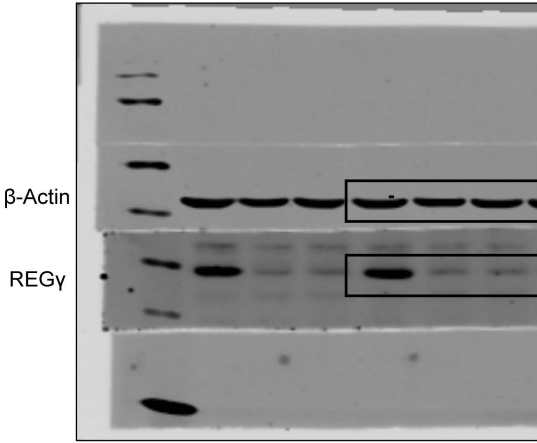

Fig. 2O

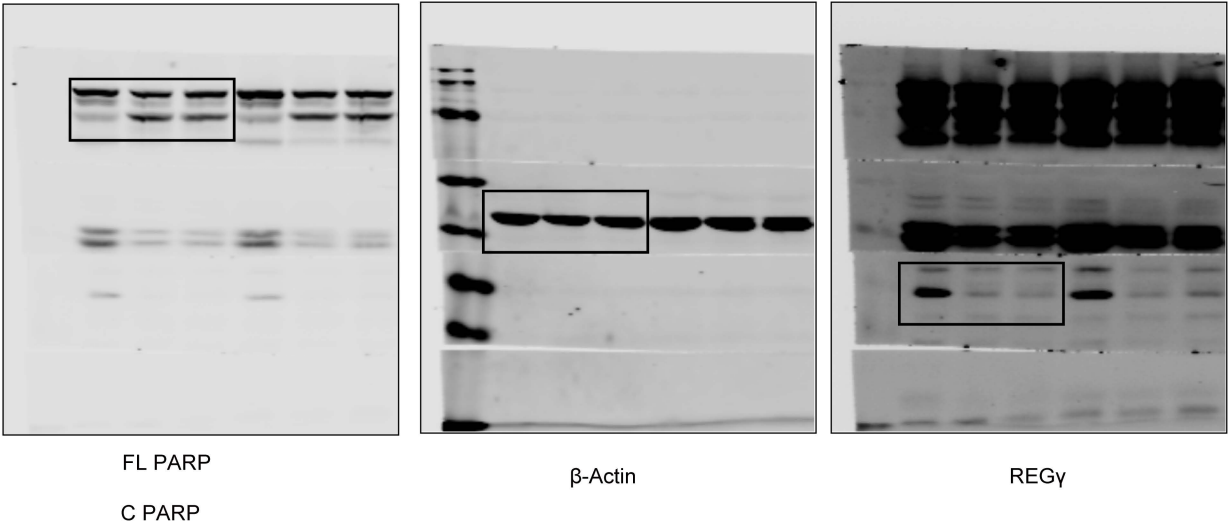

Fig. 2P

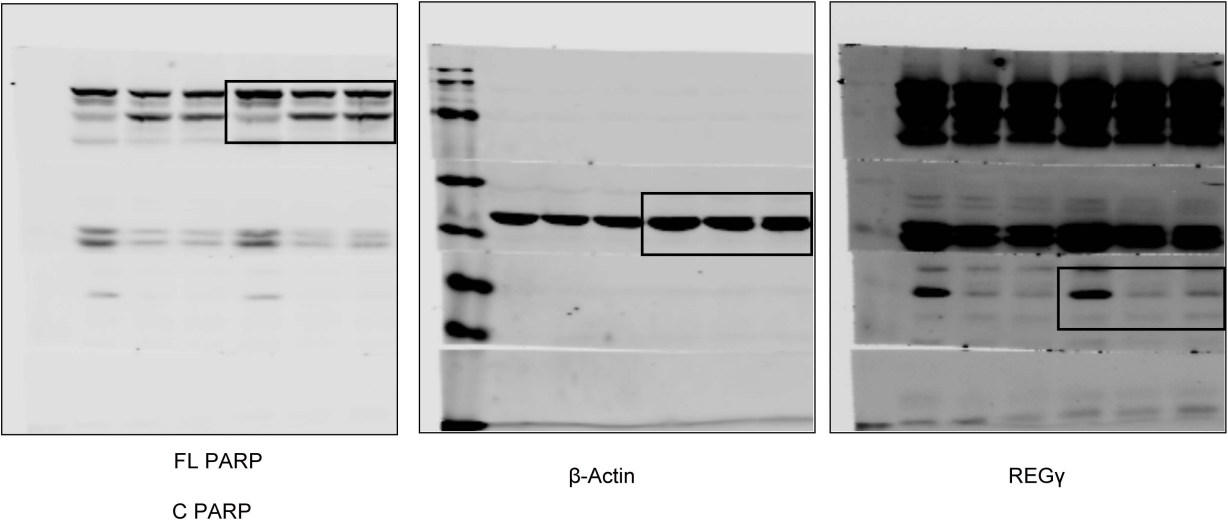

Fig. 4E

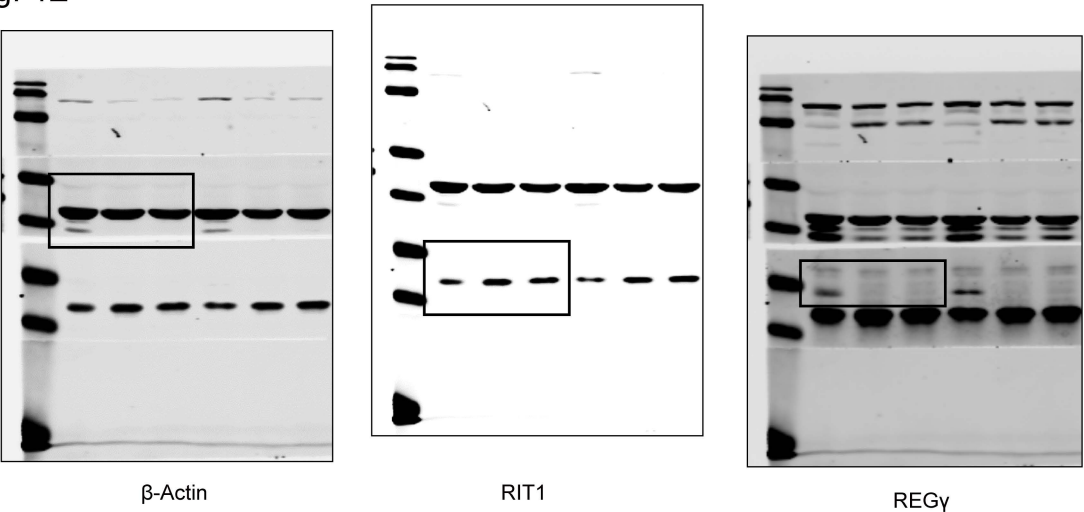

Fig. 4G

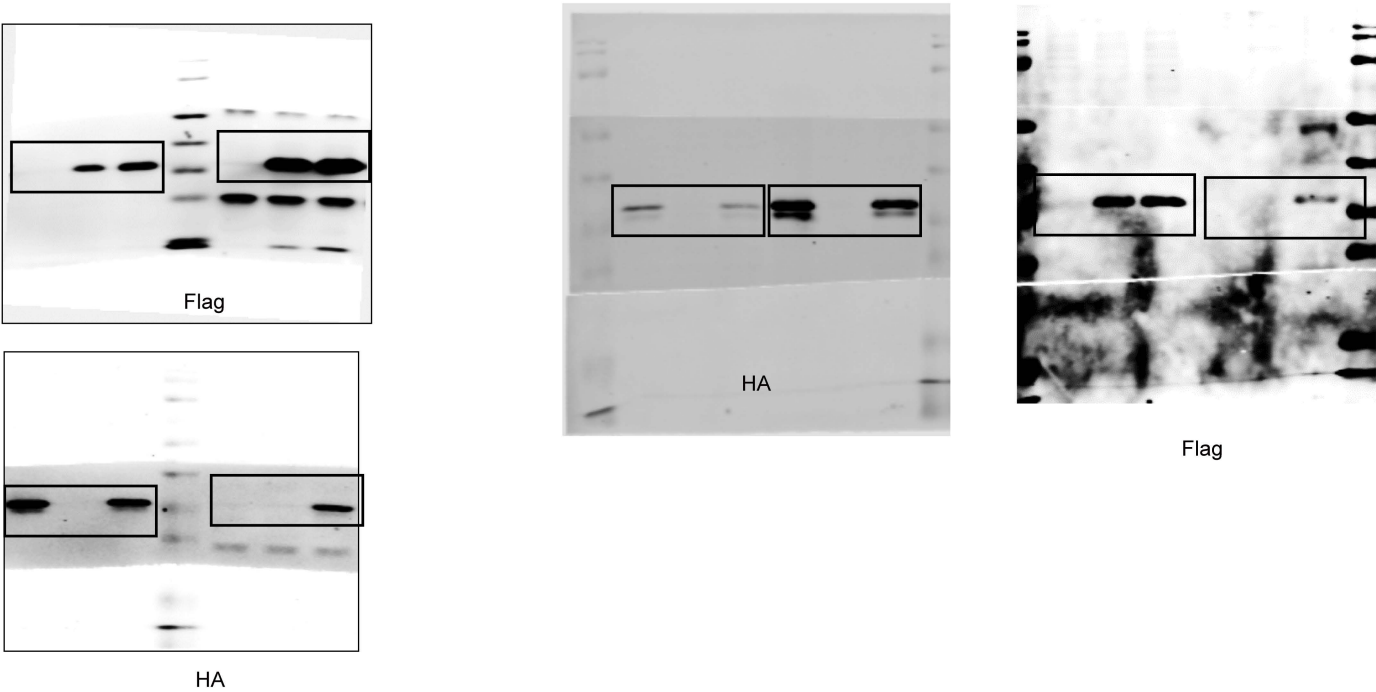

Fig. 4H

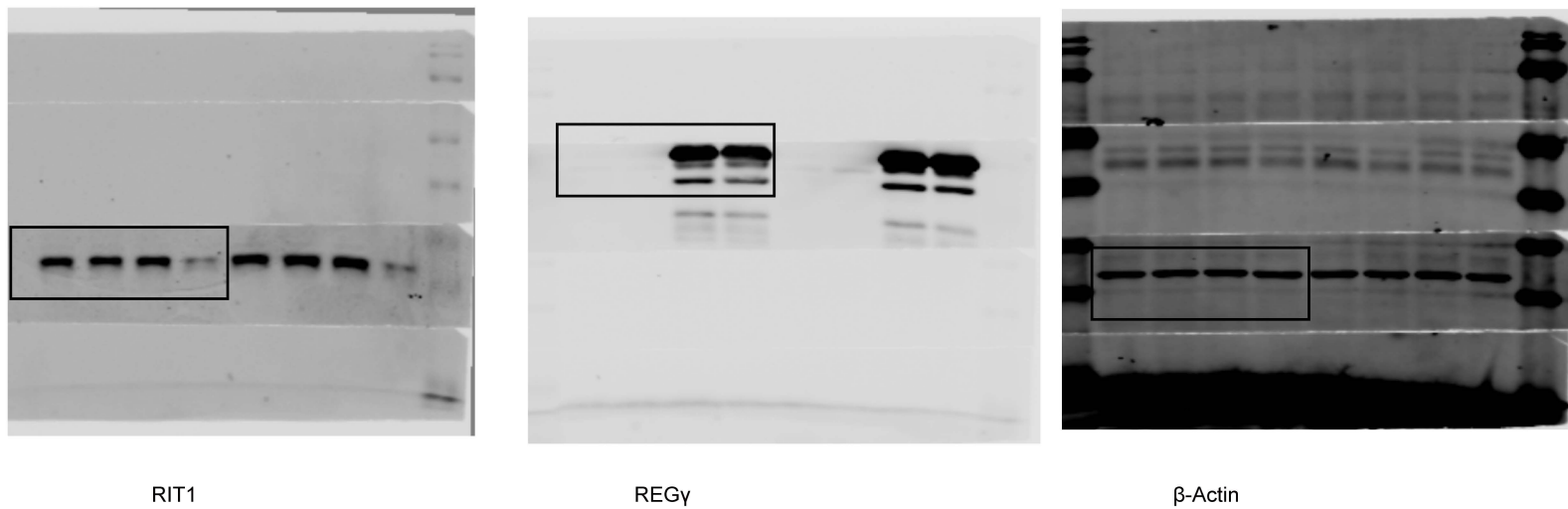

Fig. 4J

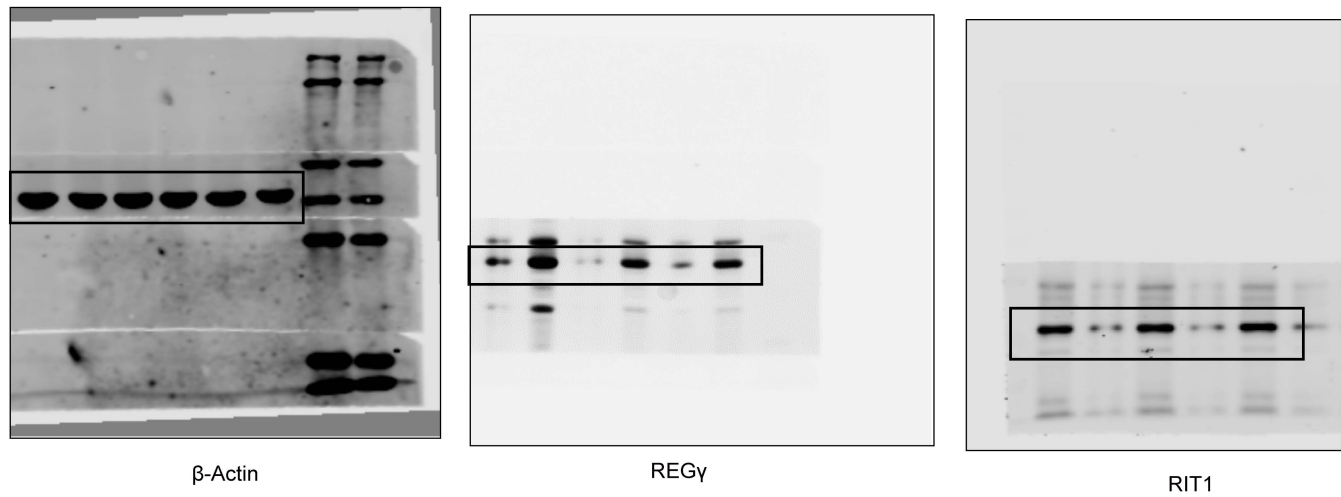

Fig. 5A

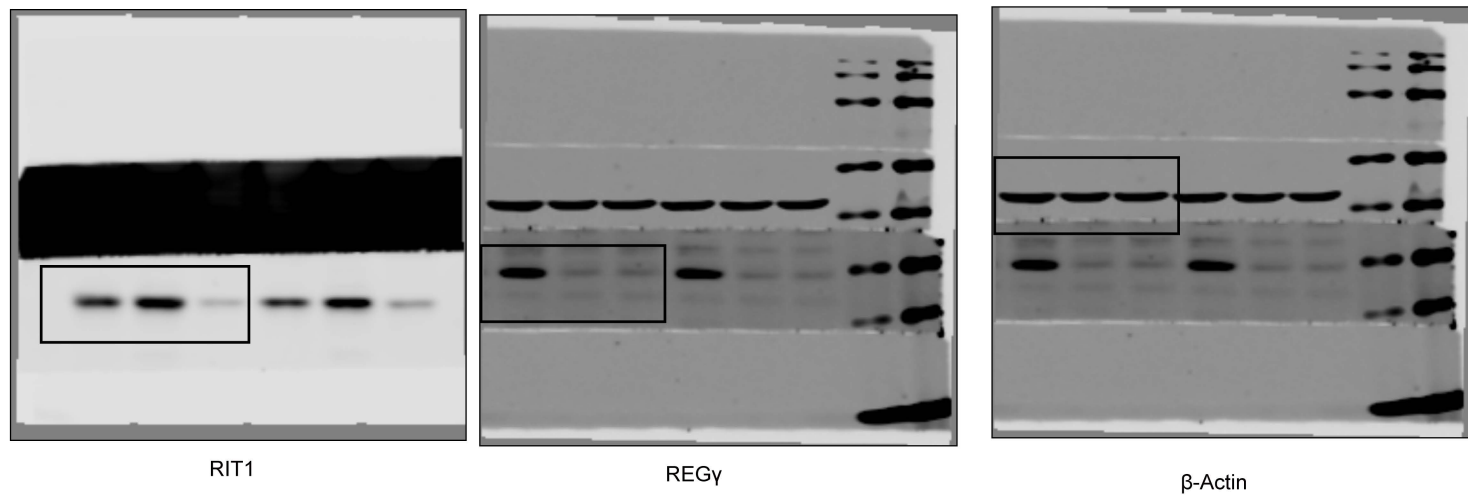

Fig. 5D

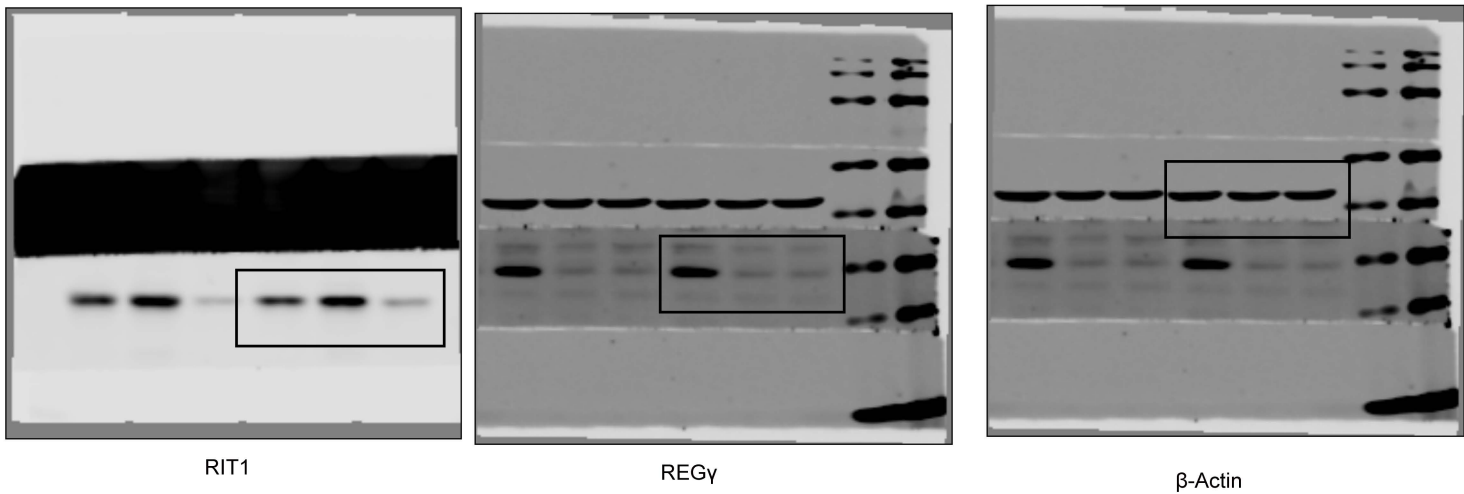

Fig. 5K

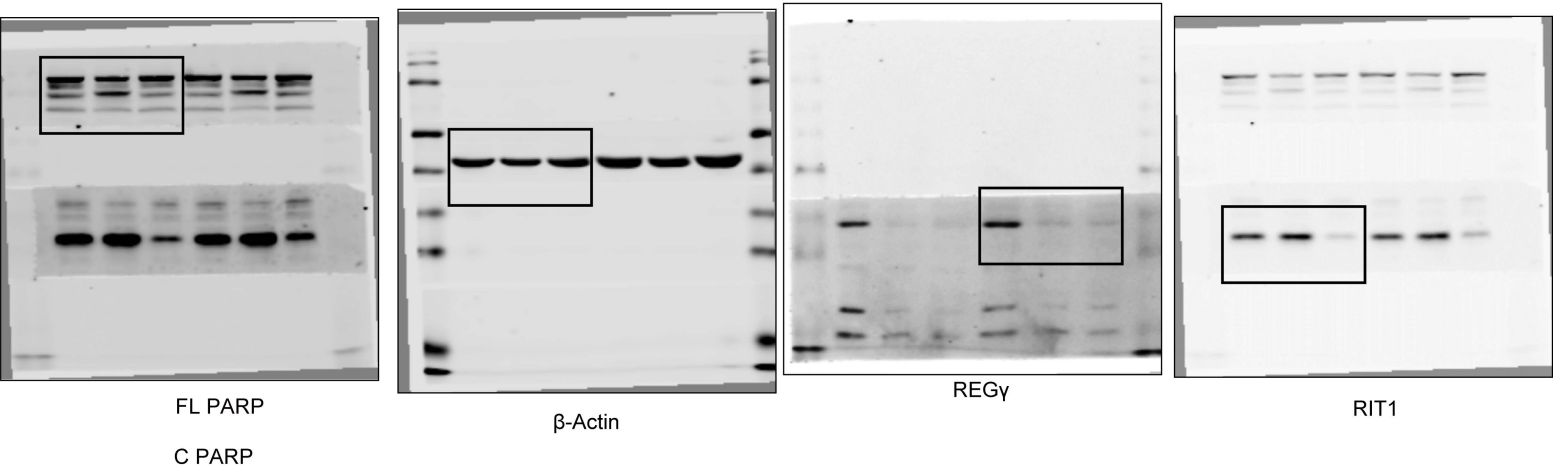

Fig. 5M

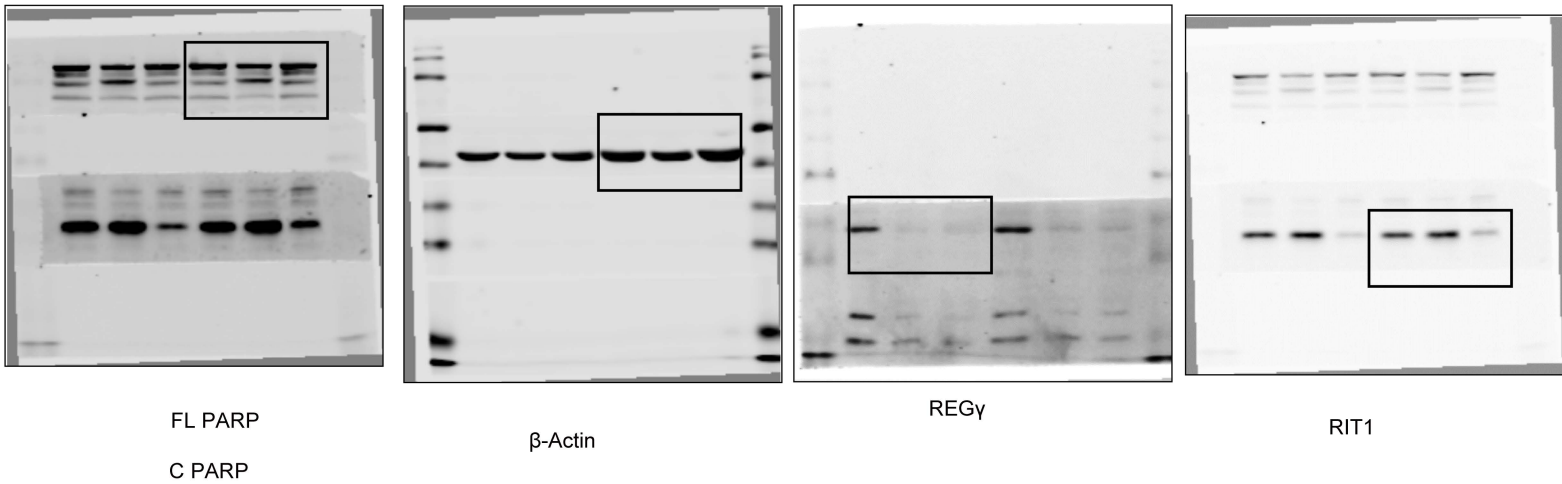

Fig. 6C

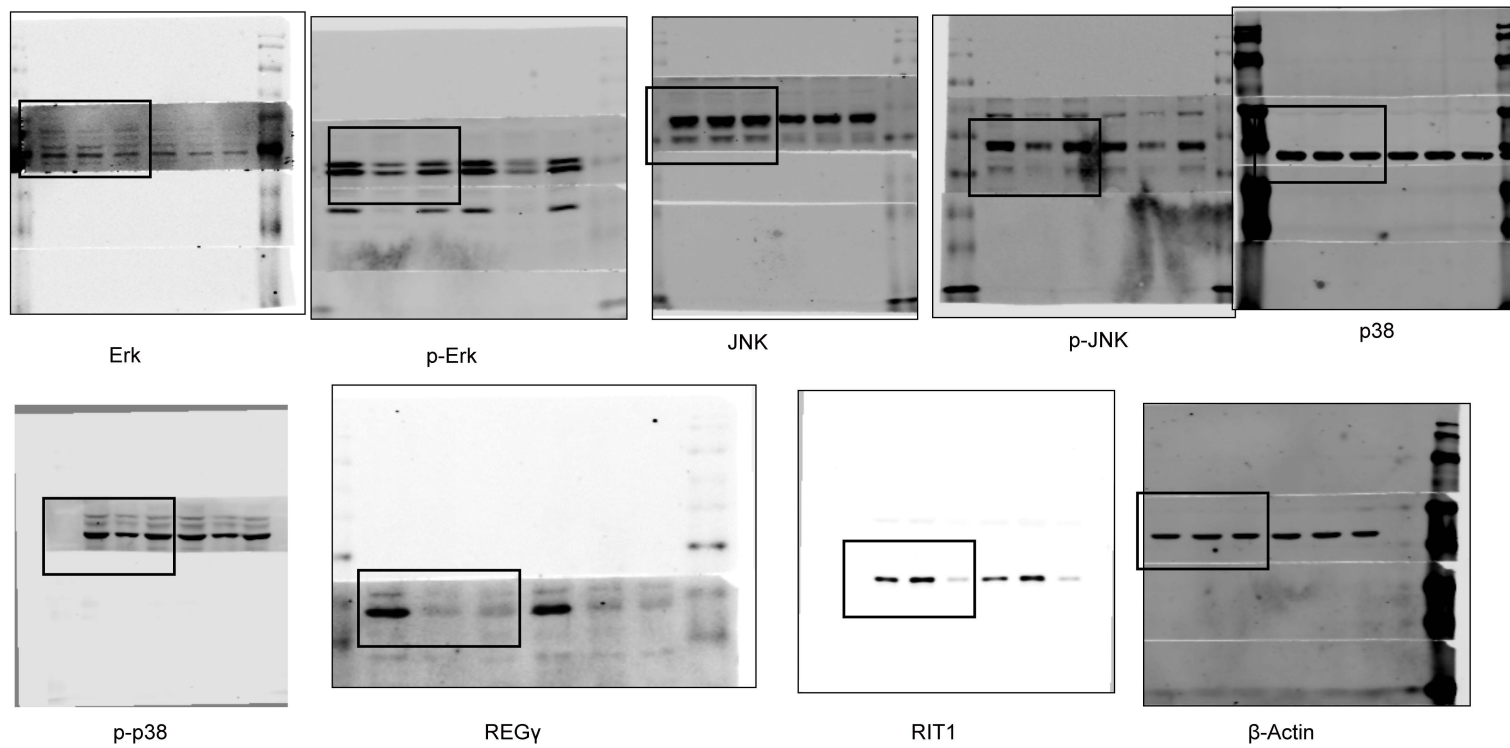

Fig. 6F

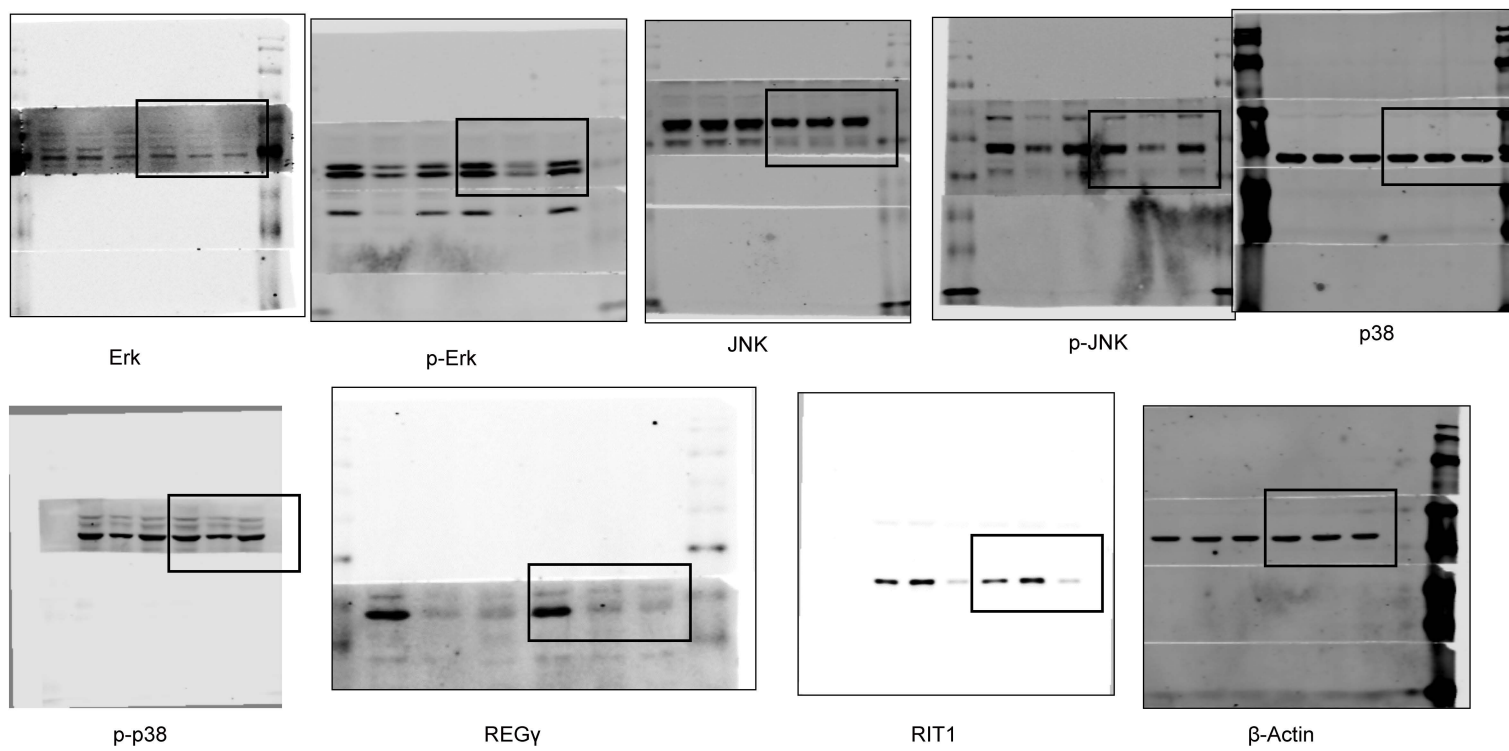

Fig. 6I

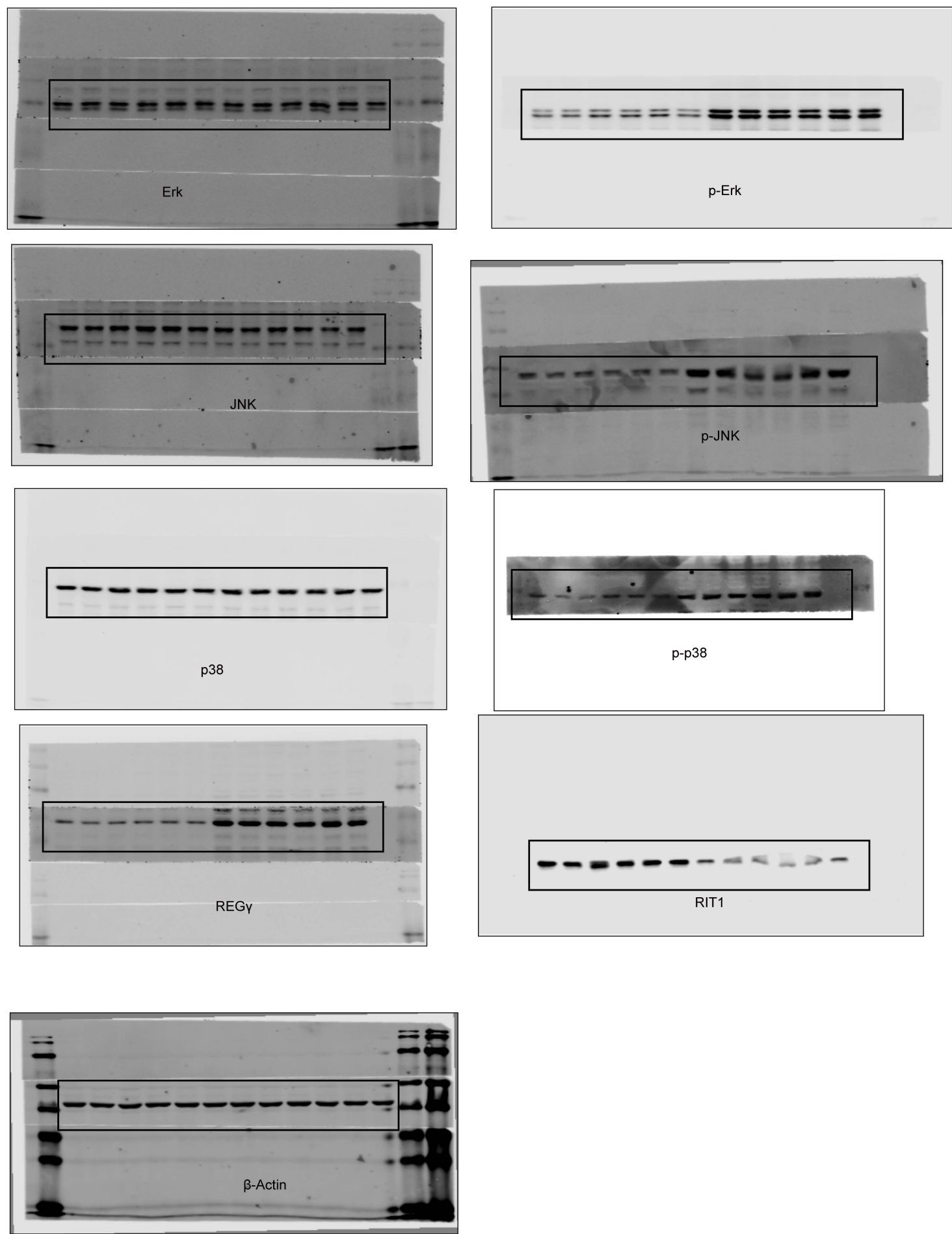

Fig. 7E

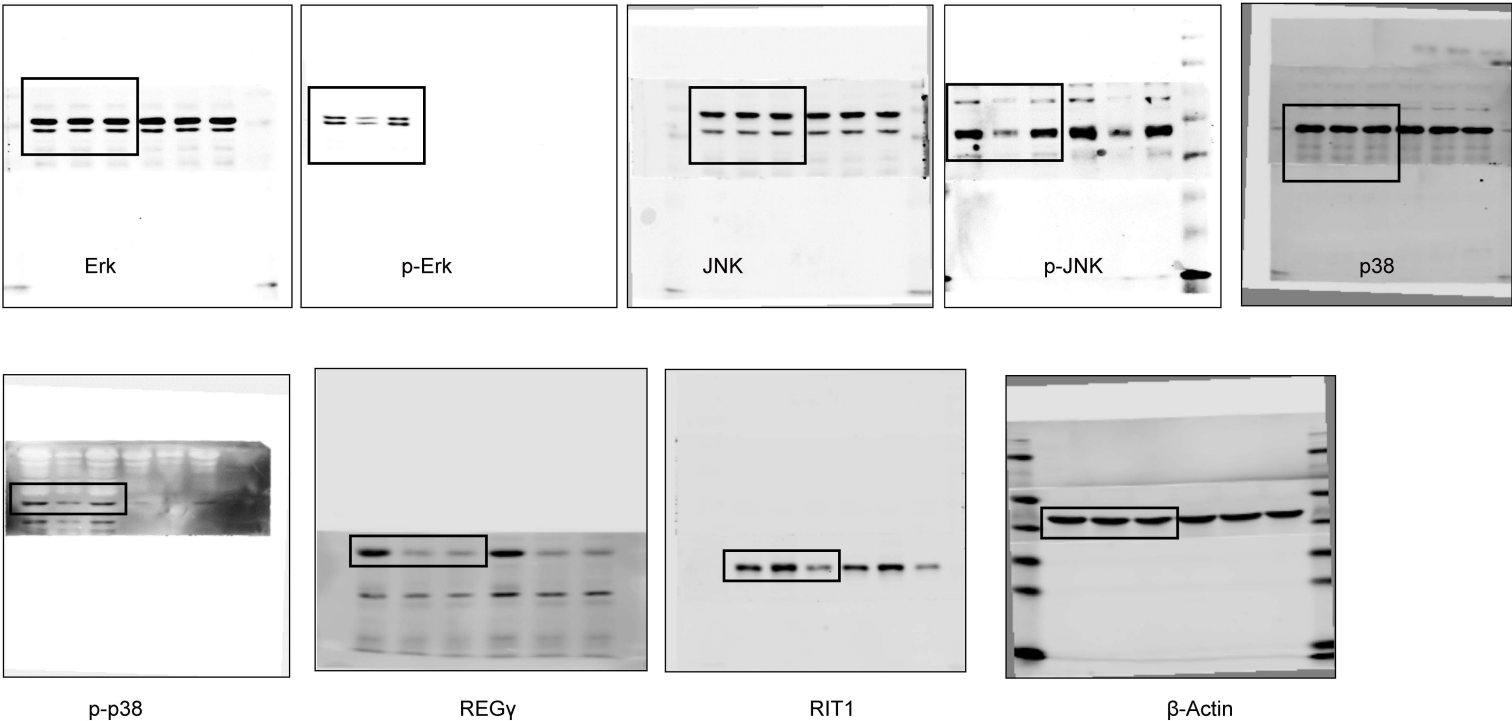

Fig. 7H

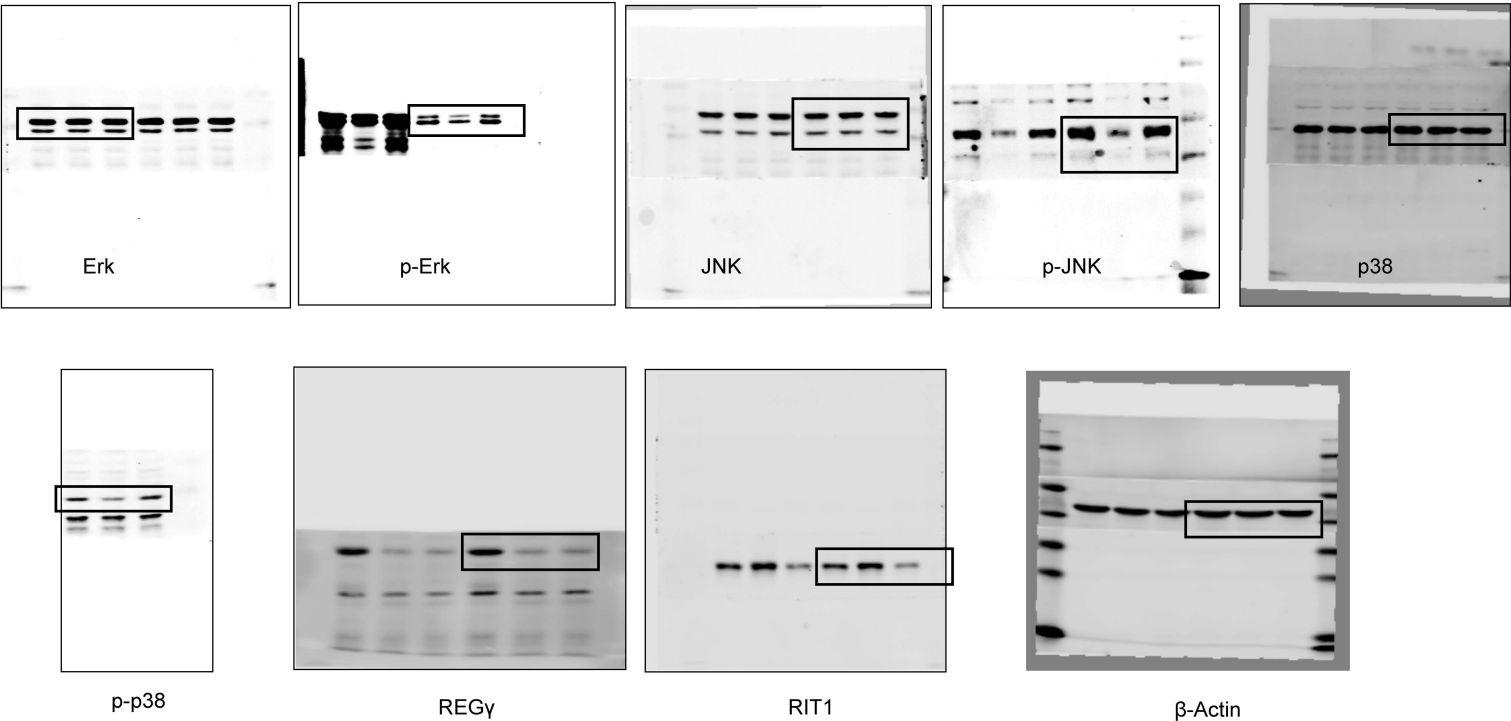

Supplement: Supplementary file 2 — Uncropped original western blots [file 41419_2025_8092_MOESM2_ESM.pdf]
